# Nonclinical Evaluation of Novel Cationically Modified Polysaccharide Antidotes for Unfractionated Heparin

**DOI:** 10.1371/journal.pone.0119486

**Published:** 2015-03-17

**Authors:** Bartlomiej Kalaska, Kamil Kaminski, Emilia Sokolowska, Dominik Czaplicki, Monika Kujdowicz, Krystyna Stalinska, Joanna Bereta, Krzysztof Szczubialka, Dariusz Pawlak, Maria Nowakowska, Andrzej Mogielnicki

**Affiliations:** 1 Department of Pharmacodynamics, Medical University of Bialystok, Bialystok, Poland; 2 Faculty of Chemistry, Jagiellonian University, Krakow, Poland; 3 Department of Cell Biochemistry, Faculty of Biochemistry, Biophysics and Biotechnology, Jagiellonian University, Krakow, Poland; Instituto Butantan, BRAZIL

## Abstract

Protamine, the only registered antidote of unfractionated heparin (UFH), may produce a number of adverse effects, such as anaphylactic shock or serious hypotension. We aimed to develop an alternative UFH antidote as efficient as protamine, but safer and easier to produce. As a starting material, we have chosen generally non-toxic, biocompatible, widely available, inexpensive, and easy to functionalize polysaccharides. Our approach was to synthesize, purify and characterize cationic derivatives of dextran, hydroxypropylcellulose, pullulan and γ-cyclodextrin, then to screen them for potential heparin-reversal activity using an *in vitro* assay and finally examine efficacy and safety of the most active polymers in Wistar rat and BALB/c mouse models of experimentally induced arterial and venous thrombosis. Efficacy studies included the measurement of thrombus formation, activated partial thromboplastin time, bleeding time, and anti-factor Xa activity; safety studies included the measurement of hemodynamic, hematologic and immunologic parameters. Linear, high molecular weight dextran substituted with glycidyltrimethylammonium chloride groups at a ratio of 0.65 per glucose unit (Dex40-GTMAC3) is the most potent and the safest UFH inhibitor showing activity comparable to that of protamine while possessing lower immunogenicity. Cationic polysaccharides of various structures neutralize UFH. Dex40-GTMAC3 is a promising and potentially better UFH antidote than protamine.

## Introduction

Although many new antithrombotic agents were introduced in the last few years, unfractionated heparin (UFH), an anionic polysaccharide, remains a key drug inhibiting blood coagulation in case of emergency. It enables open-heart surgery by preventing blood clotting in the heart-lung machine (cardiopulmonary bypass) oxygenating and supplying blood to the main body organs. After surgery almost all patients have to receive protamine: a cationic protein inactivating heparin and restoring coagulation; most would probably bleed to death without this antidote [1].

Kimmel *et al*. [2] predicted that 7% of these patients may die anyway because of serious side effects of protamine, such as anaphylactic shock, pulmonary vasoconstriction or systemic hypotension [3–6]. According to reports the incidence of protamine-related anaphylaxis varies from 0.2 to 10% [4,6]. In 2003 666,000 open-heart surgeries and 470,000 Coronary Artery Bypass Graft (CABG) procedures were performed within the USA [7]. Although in 2010 these numbers decreased by 15% due to more frequent percutaneous interventions [8], protamine usage increased in vascular surgery, such as fistula placement, abdominal aortic aneurysm repair, and other open abdominal surgeries, or transcatheter aortic valve implantations [9]. Thus, in the USA alone more than one thousand life-threatening complications a year could be attributed to the toxicity of protamine [8]. Allergy to fish may predispose to this acute response, because commercial protamine originates from sperm of salmon fished around Japan [10]. Since protamine is also used to slow down absorption and to prolong action of some insulins, diabetic patients might be prone to fatal allergic reactions [10,11]. European Medicines Agency alerted about potential supply shortage of protamine after Fukushima nuclear plant disaster. Obviously, because of the problematic supply source of protamine and safety reasons, a new heparin antidote is strongly desired. The quest for a safer alternative to protamine has a long history and has been exhaustively described in a recent review [12]. So far the pharmaceutical companies have suspended the development of a number of UFH antidotes, for example L-lysine [13], due to expensive large-scale synthesis. Thus, as a starting material for the synthesis of cationic polymers, we chose generally non-toxic, biocompatible, widely available, inexpensive, and easy to functionalize polysaccharides. We had previously explored the mechanism of UFH binding by modified polysaccharides such as chitosan [14,15], dextran [16,17], and hydroxypropylcellulose [16]. Their cationic groups attached to the polysaccharide chain bind to the anionic groups attached to the UFH chain and form inactive polycation-UFH complex.

In the present study we aimed to develop a new UFH antidote with full inhibitory potency, but safer and easier to produce than protamine. First, our team—consisting of chemists, pharmacologists and immunologists—synthesized and characterized several positively-charged polymers, whose structure, like that of heparin, is based on a polysaccharide main chain. The novel molecules originated from dextran, hydroxypropylcellulose, pullulan or γ-cyclodextrin and differed in the molecular weight, architecture and cationic charge density. Then, based on *in vitro* UFH binding assay we selected the most promising polymers and, finally, we compared the efficacy and safety of the most active agents in the animal models of thrombosis. We selected the most potent and safe heparin antidote using rat model of electrically-induced arterial thrombosis [18], which we found previously to be suitable for testing antithrombotic and anticoagulative effects of various agents [17,19–24]. We also evaluated immunogenic properties of selected novel polymers and compared them with protamine in a repeated-dose animal study.

## Materials and Methods

### Animals

Animals were purchased from and housed in the Centre of Experimental Medicine of Medical University of Bialystok in specific pathogen free conditions according to Good Laboratory Practice rules. 166 male Wistar rats and 45 BALB/c mice were used in all experiments. Animals were housed with a 12 h light/dark cycle in temperature (22 ± 2°C) and humidity (55 ± 5%) controlled room, grouped cages as appropriate, and allowed to have ad libitum access to sterilized tap water and a standard chow (Ssniff R-Z V1324). The animals’ health status was monitored throughout the experiments by a health surveillance programme according to Federation of European Laboratory Animal Science Associations (FELASA) guidelines. The rats and mice were free of all viral, bacterial, and parasitic pathogens listed in the FELASA recommendations. All the procedures involving animals and their care were approved by Local Ethical Committee on Animal Testing at the Medical University of Bialystok (Permit Numbers 28/2012 and 15/2013) and by First Local Ethical Committee on Animal Testing at the Jagiellonian University in Krakow (Permit Number 92/2012) and conducted in accordance with ARRIVE guidelines [25], directive 2010/63/EU of the European Parliament and of the Council on the protection of animals used for scientific purposes and the national laws. Procedures were conducted in the light phase of cycle in the surgical room of our laboratory. All animals were euthanized by exsanguination at the end of experiments.

### Chemicals and drugs

Dextran (Dex40, Mw = 40 kDa from *Leuconostoc* spp.; Dex6, Mw = 6 kDa from *Leuconostoc* spp.), hydroxypropylcellulose (HPC, Mn 10 kDa, Mw 80 kDa), pullulan (Pul, 200 kDa, from *Aureobasidium pullulans*), γ-cyclodextrin (GCD, ≥90.0% cyclodextrin basis (HPLC), N-(3-dimethylaminopropyl)-N′-ethylcarbodiimide hydrochloride (EDC, ≥99.0%), N-hydroxysuccinimide (NHS, 98%), l-arginine (reagent grade, ≥98% TLC), allylamine (≥99%), spermine (Spm, >97%), 2,2′-azobis(2-methylpropionamidine) dihydrochloride (AAPH, 97%), N,N'-carbonyldiimidazole (CDI, reagent grade), N-acrylamidopropyl-N,N,N-trimethylammonium chloride (APTMAC, 75 wt% solution in water, stabilized with 3000 ppm MEHQ), glycidyltrimethylammonium chloride (GTMAC, technical ≥90%), benzoyl peroxide (BPO, 75% remainder water Luperox A75), heparin sodium salt from bovine intestinal mucosa (UFH), protamine (protamine sulfate salt from salmon, grade X), N,N-dimethylformamide (DMF, analytical grade), dimethyl sulfoxide (DMSO, analytical grade), trisodium citrate (≥99%) were purchased from Sigma-Aldrich (Germany). Dex1 (Mw = 1 kDa from *Leuconostoc* spp.) was purchased from Pharmacosmos (Denmark). Azure A chloride (Fluka standard) was purchased from Fluka (Switzerland). Acetone, ethanol 96%, methanol, potassium chloride, potassium dihydrogen phosphate, disodium hydrogen phosphate, sodium chloride, NaOH, were all analytical grade and purchased from POCh (Poland). Routine laboratory reagents to determine activated partial thromboplastin time (aPTT) in plasma were purchased from Bio-Ksel (Poland). Anti-Xa assay kits were purchased from Sekisui Diagnostics (USA). Pentobarbital, ketamine, and xylazine were purchased from Biovet (Poland).

### Polymer synthesis and characteristics

Polysaccharides substituted with GTMAC were synthesized using the general procedure described previously [16]. All the details of polymers synthesis and solubility are presented in S1 File. UV-Vis absorption spectra were recorded using an HP8452A diode-array spectrophotometer in 1-cm optical path quartz cells. The dimensions of the aggregates in aqueous suspensions were determined using Malvern Instruments Ltd Nano ZetaSizer. FTIR spectra were obtained using a Bruker IFS 48 spectrometer. NMR spectra were measured in D_2_O using a Bruker AMX 500 spectrometer. GPC analyses were performed using a Waters GPC system equipped with a bank of three columns (PL Aquagel-OH 30, 40, and 60) and tandem PDA/RI detectors. The eluent was 0.1 M NaCl, flow rate was 0.6 ml·min^-1^, sample volume was 150 μl, and the concentration of polymers was 5 g·l^-1^.

### Binding of UFH by the cationic polymers

**Fig 1:**
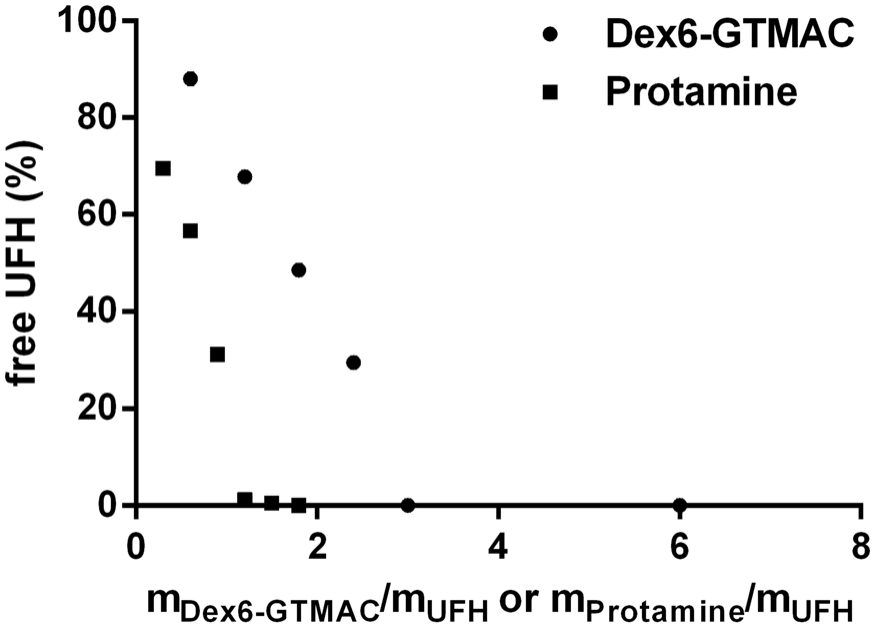
Binding of UFH by the cationic polymers. Dependence of free UFH concentration on the concentration of Dex6-GTMAC expressed as the ratio of the Dex6-GTMAC mass and total UFH mass. Data for protamine are shown for comparison.

The ability of the studied substances to bind UFH was assessed with a colorimetric method using Azure A, a cationic dye, as described previously [14,15]. Briefly, UFH chains, when present in the solution of Azure A, complex the dye molecules, which then form aggregates absorbing at 513 nm, while the monomeric form of the dye (in the absence of UFH) absorbs at 630 nm. Added polymer disrupts dye aggregates partially (or fully in the case of complete binding of UFH) and increases 630 nm and decreases 513 nm absorption band intensity. A representative example of the plot showing the dependence of free (i.e., uncomplexed and therefore possessing anticoagulant activity) UFH concentration on the cationic polymer concentration, expressed as the ratio of the cationic polymer mass and total UFH mass, is shown in Fig. 1.

### Experimental arterial thrombosis in rats

84 male Wistar rats weighing 216 ± 10.8 g (no significant difference between groups) were randomly divided into 10 groups (8–10 per experimental group), anesthetized by an intraperitoneal injection of pentobarbital (45 mg·kg^-1^ b.w.) and placed in a supine position on a heated operation table. UFH was administered into right femoral vein in a volume not exceeding 0.5 ml·kg^-1^ b.w. 10 min before the induction of thrombosis in dose of 300 U·kg^-1^ b.w. alone or followed (3 min) by intravenous (iv) administration of Dex40-GTMAC2 (4.2 mg·kg^-1^ b.w.), Dex40-GTMAC3 (2.5 and 7.5 mg·kg^-1^ b.w.), Dex6-GTMAC (9.6 mg·kg^-1^ b.w.), GCD-GTMAC2 (10.8 mg·kg^-1^ b.w.), or protamine (3 mg·kg^-1^ b.w.). Vehicle (PBS) treated animals served as a control group. Additionally, UFH was administered into right femoral vein of 16 male Wistar rats, weighing 209.7 ± 9.9 g in dose of 900 U·kg^-1^ b.w. alone or followed by iv administration of Dex40-GTMAC3 (22.5 mg·kg^-1^ b.w.). Dex40-GTMAC3 was also administered alone in dose of 7.5 mg·kg^-1^ b.w. Arterial thrombosis was induced by electrical stimulation of the common carotid artery, according to methods previously described [18,26]. Briefly, the anode, a stainless steel L-shaped wire, was inserted under the artery and connected to a circuit with a constant current generator. The cathode was a subcutaneous metal needle attached to the hindlimb. The stimulation (1 mA) took 10 min. 45 min after stimulation the segment of the common carotid artery with the formed thrombus was clipped at both sides, dissected opened lengthwise and the thrombus was completely removed, air-dried in 37°C and weighed 24 h after the end of experiment. Thrombus weight was our primary endpoint, whereas coagulation markers measured in plasma served as secondary endpoints. After removal of formed thrombus blood samples were taken from the heart. The blood samples were drawn into 3.13% trisodium citrate in a volume ratio of 9:1, then centrifuged at 3500 x g at 4°C for 20 min and plasma was deep-frozen (-70°C) in aliquots of 1 ml until further assays could be performed. A time-line diagram of this experiment was presented in our previous publication [17].

### Experimental venous thrombosis in mice

25 male BALB/c mice weighing 24.2 ± 0.2 g (no significant difference between groups) and aged 8–10 weeks were randomly divided into 4 groups (5–7 per experimental group). Animals were anesthetized with a mixture of ketamine (125 mg·kg^-1^ b.w.) and xylazine (12.5 mg·kg^-1^ b.w.). After the anesthesia animals were heparinized with 300 U·kg^-1^ b.w. (50 μl iv into tail vein). Three minutes after injection, UFH was neutralized with protamine (3 mg·kg^-1^ b.w.) or Dex40-GTMAC3 (7.5 mg·kg^-1^ b.w.). The Electrolytic Inferior Vena Cava Model was performed as previously described [27]. Briefly, the abdomen was opened, and all inferior vena cava side branches were carefully ligated. The 25G stainless-steel needle was inserted into the exposed caudal vena cava and connected to a current source of 25 μA over 15 minutes (anode). After 15 minutes the needle was removed and abdomen was closed in a two-layer fashion. After six hours the animals were reanesthetized and the abdomen was reopened, the vena cava was carefully dissected and inspected for the presence of thrombus. The thrombus was kept at 37°C and after 24 hours its dry weight was measured.

### Bleeding time in rats

The tail bleeding time was measured after removal of formed thrombus using a standardized Simplate device (Simplate II, Organon Teknika Corp., USA). The device makes two incisions 5 mm long and 1 mm deep on the dorsal part of the tail between 9 and 11 cm from the tip, taking care to avoid large veins. Immediately after injury, the tail was placed into the cylinder with isotonic saline at 37°C. Bleeding time was measured from the moment the tail was surgically cut until bleeding stopped completely (no rebleeding within 30 s). If bleeding was still present at the end of the 300 s observation period, a value of 300 s was ascribed for the sake of statistical analysis. Bleeding time was expressed in seconds.

### aPTT measurement in rats and mice

The aPTT was measured in plasma collected from thrombotic rats or mice as a basic coagulation parameter used for monitoring of UFH action. Additionally, Dex40-GTMAC3 was assayed for potential anticoagulant activity by measuring of aPTT according to a modified procedure of Byun et al. [28]. The blood samples were taken from the heart of 5 male Wistar rats, and were drawn into 3.13% trisodium citrate in a volume ratio of 9:1, then centrifuged at 3500 × g at 4°C for 20 min and 200 μl of pooled plasma was mixed with 20 μl of a solution containing increasing concentrations of Dex40-GTMAC3. The aPTT values were automatically determined with an optical method (Coag-Chrom 3003; Bio-ksel, Poland) adding routine laboratory reagents to collected animal plasma.

### Anti-factor Xa activity in rats

Anti-factor Xa activity was analyzed with ELISA technique at 25°C using a microplate reader (Dynex Tech., USA) according to the kit manufacturer instructions.

### Blood cell count in rats

Blood cell count was assessed with an Animal Blood Counter (ABC Vet, Horiba, Germany) according to the manufacturer directions.

### Blood pressure measurement in rats

The mean blood pressure (MBP) was measured directly through a cannula filled with UFH solution (150 U·ml^-1^), placed in the left common carotid artery and connected to the pressure transducer (Plugsys, Transonics System, USA) in anaesthetized Wistar rats weighing 239.7 ± 7.2 g (no significant difference between groups), as described previously [29]. 74 Wistar rats were randomly divided into 16 groups (4–6 per experimental group). The schedule of drug administration was the same as in arterial thrombosis experiment. Additionally, polymers were administered alone in 3 times higher doses.

### Immunization experiments

20 female BALB/c mice weighing 26.3 ± 0.5 g (no significant difference between groups) and aged 8–10 weeks were randomly divided into 4 groups (5 per experimental group). Animals were anesthetized with a mixture of ketamine (125 mg·kg^-1^ b.w.) and xylazine (12.5 mg·kg^-1^ b.w.) and were heparinized with 150 U·kg^-1^ b.w. (50 μl iv into tail vein). Three minutes after injection, UFH was neutralized with protamine (1.5 mg·kg^-1^ b.w.), or Dex40-GTMAC2 (6.25 mg·kg^-1^ b.w.), or Dex40-GTMAC3 (3.75 mg·kg^-1^ b.w.). All antidotes were injected iv into tail vein in 50 μl of saline, while the control group (UFH) was not administered any antidote. The heparinization/neutralization regimen was repeated 5 times, once every week (days 1, 8, 15, 22, and 29). Blood samples were collected from the tail vein of each mouse one day prior UFH administration and serum was isolated by centrifugation. One week after the last injection (day 36) all mice were sacrificed; final blood samples were collected and spleens of the animals were isolated for evaluation.

### Immune response evaluation

The levels of antibodies specific to protamine, Dex40-GTMAC2, and Dex40-GTMAC3 were evaluated using standard indirect ELISA. Briefly, wells of a 96-well plate (Nunc MaxiSorp) were coated O/N at RT with 50 μl of antigen solution (25 μg·ml^-1^ in PBS). The wells were blocked O/N with 200 μl of 1% BSA in PBS at 4°C. Serum samples were diluted in PBS, added to the wells washed with PBS, and incubated O/N at 4°C. Murine antibodies bound to the antigen were detected with horseradish peroxydase-conjugated secondary antibodies specific to mouse IgM (Sigma A8786, 1:5 000) or mouse IgG (Sigma A3673, 1:10 000). Colorimetric detection was based on TMB substrate solution (BD Biosciences) and the enzymatic reaction was stopped by adding 50 μl of 0.18 M H_2_SO_4_. ELISA signals were measured at 450 nm using VERSAmax microplate reader (Molecular Devices). All sera were tested on each antigen (protamine, Dex40-GTMAC2, and Dex40-GTMAC3) to detect potential cross-reactivity.

### Statistical analysis

In the study, n refers to number of animals in each experimental group. For each test, the experimental unit was an individual animal. We choose the minimal number of animals to detect differences between each group basing on our and others experience using these procedures. The data are shown as mean ± SD and analyzed using the non-parametric Mann-Whitney test. P values less than 0.05 were considered significant, less than 0.01 highly significant and less than 0.001 extremely significant.

## Results

### Characteristics of the synthesized polymers

Table 1 shows characteristics of obtained polymers. Their structure varied in the type of: modified polysaccharide (Dex 1, 6 or 40 kDA, Pul, HPC or GCD), type of cationic group (GTMAC, APTMAC, Spm, PAH or PAH-ARG), degree of substitution, and charge expressed as zeta potential. All the details of elemental analysis, ^1^H nuclear magnetic resonance (NMR) and Fourier transform infrared (FT-IR) spectroscopy measurements are presented in S2 File.

**Table 1:** Characteristics of the polymers.

Polymer	MW (kDa)^a^	ξ^b^ (mV)	Degree of substitution^c^	UFH binding^d^
**Dex1-GTMAC**	1	-	0.45	no binding
**Dex6-GTMAC**	6	3.0 ± 0.6	0.59	2.7
**Dex40-GTMAC1**	40	~0	0.10	insufficient binding^e^
**Dex40-GTMAC2**	40	1.2 ± 0.4	0.50	2.7
**Dex40-GTMAC3**	40	3.2 ± 0.6	0.65	1.6
**Dex40-APTMAC**	40	1.0 ± 0.7	0.42	insufficient binding^e^
**Dex40-Spm**	40	1.9 ± 0.3	0.71	1.0
**Dex40-PAH**	40	0–2^f^	1.17	insoluble
**Dex40-PAH-Arg**	40	1.2 ± 2.8	0.10^g^	2.2
**Pul-GTMAC**	200	2.6 ± 0.1	0.71	1.9
**GCD-GTMAC1**	1.297	-	0.66	no binding
**GCD-GTMAC2**	1.297	-	0.99	2.8
**HPC-APTMAC1**	80	2.8 ± 0.2	0.22	insufficient binding^e^
**HPC-APTMAC2**	80	8.5 ± 0.4	4.11	1.0

Dex—dextran, Pul—pullulan, GCD—γ-cyclodextrin, HPC—hydroxypropylcellulose, GTMAC—ammonium group of glycidyltrimethyl-ammonium chloride, APTMAC—ammonium group of N-acrylamidopropyl-N,N,N-trimethylammonium chloride, Spm—primary and secondary amine groups of N,N′-Bis(3-aminopropyl)-1,4-diaminobutane (Spermine), PAH—primary amine group of poly(allylamine hydrochloride), PAH-Arg—primary amine group of poly(allylamine hydrochloride), guanidyl group of arginine,

^a^ MW—weight average molecular weight, for GCD exact molecular weight is given

^b^ ξ—zeta potential

^c^ degree of substitution defined as the number of the cationic groups per a glucose unit, as found from elemental analysis

^d^ UFH binding—ratio of polymer mass to UFH mass required for binding 90% of UFH

^e^ the mass of the polymer required to bind UFH was at least 8 times higher than that of protamine

^f^ depending on concentration

^g^ degree of substitution defined as the number of arginine groups per PAH repeating unit

### *In vitro* UFH binding by the cationic polymers

The concentration of free UFH in the solution decreased with increasing concentration of all the synthesized polymers, except for Dex1-GTMAC and GCD-GTMAC1. Generally, the polymers with lower degree of substitution with cationic groups bound UFH weaker than protamine i.e., their higher concentration was required to achieve the same decrease in free UFH concentration. The amount of polymers necessary for binding 90% of 1 mg of UFH was used as a measure of the binding efficiency. If this value exceeded the analogous value for protamine more than eight times the compound was disqualified from further biological studies. This value was also used for the preliminary assessment of the dose of a cationic polymer required in biological tests.

### Reversal of the effects of UFH on the arterial thrombosis in rats

**Fig 2:**
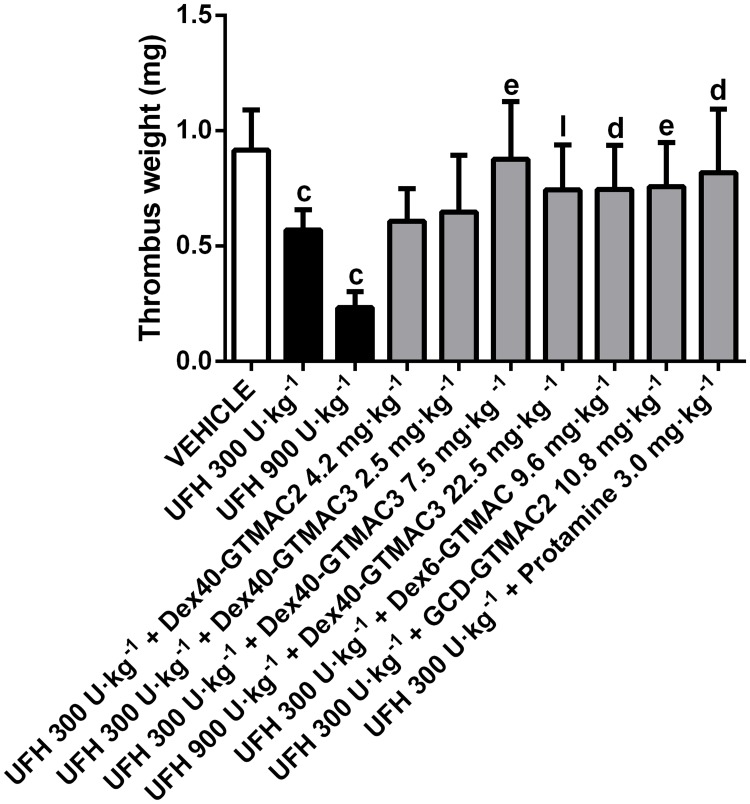
Reversal of the effects of UFH on the arterial thrombosis in rats. Dry thrombus weight in Wistar rats treated with vehicle (PBS), UFH in dose of 300 or 900 U· kg^-1^) alone or followed by Dex40-GTMAC2 (4.2 mg·kg^-1^), Dex40-GTMAC3 (2.5, 7.5 or 22.5 mg·kg^-1^), Dex6-GTMAC (9.6 mg·kg^-1^), GCD-GTMAC2 (10.8 mg·kg-1), and protamine (3.0 mg·kg^-1^), c-P<0.001 vs. vehicle; d-P<0.05, e-P<0.01 vs. UFH 300 U·kg^-1^; l-P<0.001 vs. UFH 900 U·kg^-1^, Mann-Whitney test. Results are shown as mean ± SD, n = 8–10.

UFH dose dependently decreased the weight of arterial thrombus in rats. Studied agents reversed antithrombotic effect of UFH (Fig. 2). The polymers were administered in doses corresponding with the ratio of polymer/UFH estimated in the UFH binding assay. Dex40-GTMAC3 was the most potent and its effect was comparable to the effect of protamine. It also reversed the antithrombotic effect of UFH administered in dose of 900 U·kg^-1^ b.w. (Fig. 2). Dex40-GTMAC2 administered in dose of 4.2 mg·kg^-1^ b.w. was completely ineffective. When administered alone Dex40-GTMAC3 did not influence thrombus weight (0.98 ± 0.25 mg) in comparison to control group (0.92 ± 0.17 mg).

### Reversal of the effects of UFH on the venous thrombosis in mice

**Fig 3:**
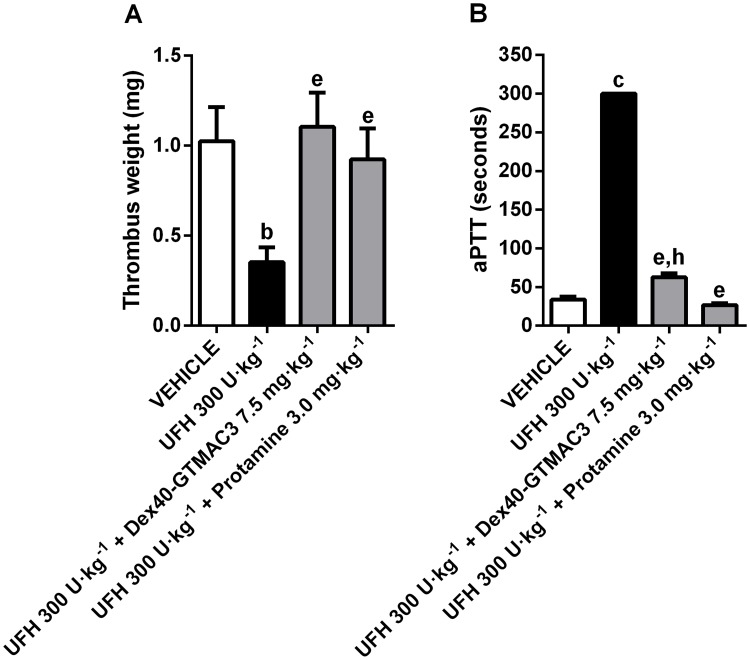
Reversal of the effects of UFH on the venous thrombosis and aPTT in mice. Dry thrombus weight **(A)** and the aPTT **(B)** in mice treated with vehicle (PBS), UFH (300 U·kg^-1^) alone or followed by Dex40-GTMAC3 (7.5 mg·kg^-1^), and protamine (3.0 mg·kg^-1^), b-P<0.01, c-P<0.001 vs. vehicle; e-P<0.01 vs. UFH 300 U·kg^-1^; h-P<0.01 vs. UFH 300 U·kg^-1^ + Protamine 3.0 mg·kg^-1^, Mann-Whitney test. Results are shown as mean ± SD n = 5–7.

Similarly to the results in the model of the arterial thrombosis in rats, UFH significantly decreased thrombus weight and prolonged aPTT in mice developing electrically induced venous thrombosis. We confirmed the ability of Dex40-GTMAC3 to reverse antithrombotic and anticoagulant effects of UFH in mice (Fig. 3).

### Influence of the cationic polymers on aPTT

**Fig 4:**
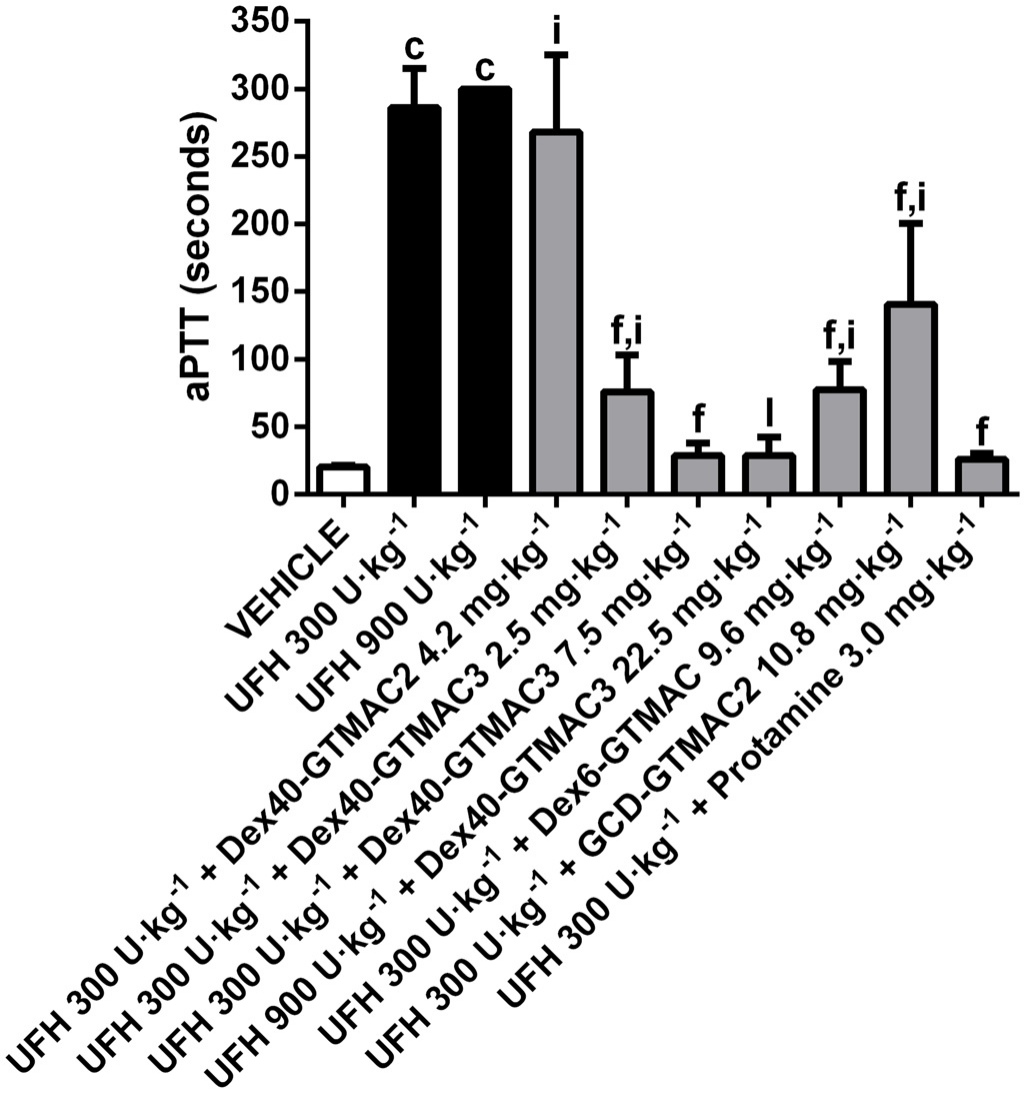
Influence of the cationic polymers on aPTT in rats. aPTT in Wistar rats treated with vehicle (PBS), UFH (300 U·kg^-1^) alone or followed by Dex40-GTMAC2 (4.2 mg·kg^-1^), Dex40-GTMAC3 (2.5 and 7.5 mg·kg^-1^), Dex6-GTMAC (9.6 mg·kg^-1^), GCD-GTMAC2 (10.8 mg·kg^-1^), and protamine (3.0 mg·kg^-1^), c-P<0.001 vs. vehicle; f-P<0.001 vs. UFH 300 U·kg^-1^; i-P<0.001 vs. UFH 300 U·kg^-1^ + Protamine 3.0 mg·kg^-1^; l-P<0.001 vs. UFH 900 U·kg^-1^, Mann-Whitney test. Results are shown as mean ± SD, n = 8–10.

In vivo, Dex40-GTMAC3, Dex6-GTMAC, GCD-GTMAC1, and protamine significantly shortened aPTT which was prolonged by earlier administration of UFH. We found that the effect of GCD-GTMAC2 was the weakest, whereas Dex40-GTMAC3 and protamine completely reversed the effects of UFH to the control value (Fig. 4). Dex40-GTMAC3 reversed also aPTT prolonged by 900 U·kg^-1^ b.w. of UFH, and when administered alone slightly (by 30%), but significantly prolonged aPTT to 26.28 ± 6.94 seconds in comparison to 20.28 ± 1.08 seconds in control group (P<0.01).

**Fig 5:**
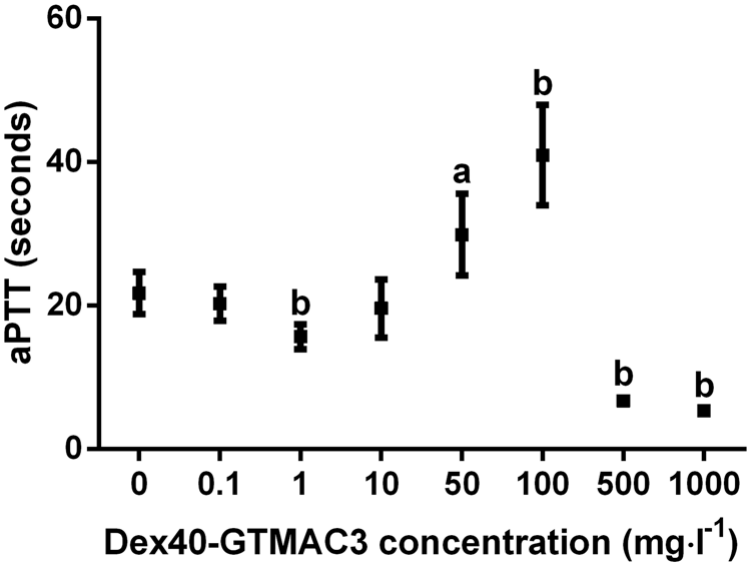
aPTT in plasma of Wistar rats mixed with different concentrations of Dex40-GTMAC. a-P<0.05, b-P<0.01 vs. vehicle, Mann-Whitney test. Results are shown as mean ± SD, n = 5.

In vitro, Dex40-GTMAC3 prolonged aPTT at higher concentration than 50 mg·l^-1^ (Fig. 5).

### Reversal of tail bleeding time by the cationic polymers

**Fig 6:**
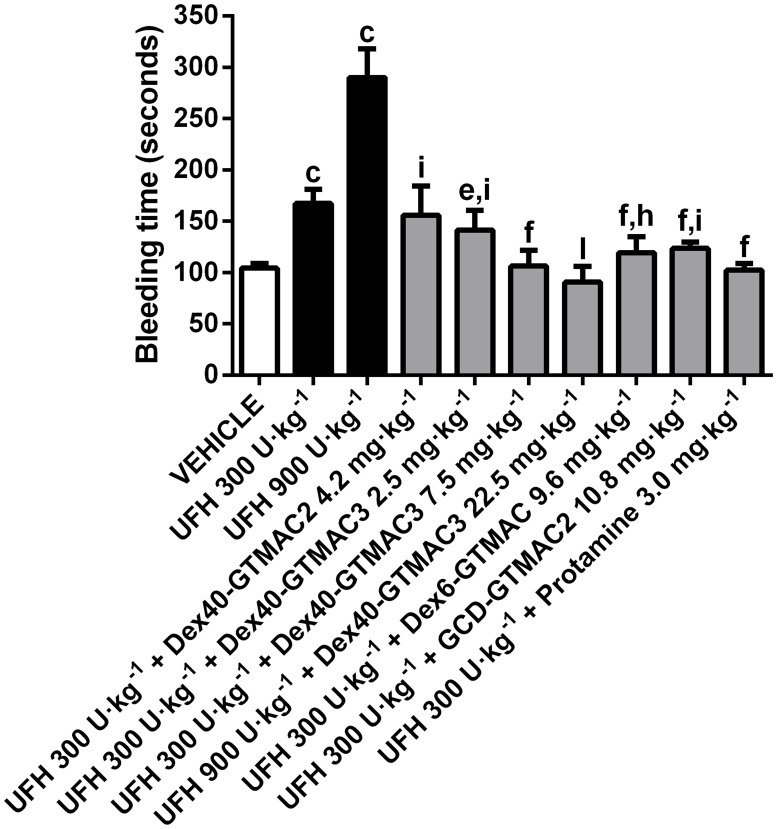
Reversal of tail bleeding time by the cationic polymers. Tail bleeding time in Wistar rats treated with vehicle (PBS), UFH in dose of 300 or 900 U·kg^-1^ alone or followed by Dex40-GTMAC2 (4.2 mg·kg^-1^), Dex40-GTMAC3 (2.5, 7.5 or 22.5 mg·kg^-1^), Dex6-GTMAC (9.6 mg·kg^-1^), GCD-GTMAC2 (10.8 mg·kg^-1^), and protamine (3.0 mg·kg^-1^), c-P<0.001 vs. vehicle; e-P<0.01, f-P<0.001 vs. UFH 300 U·kg^-1^; h-P<0.01, i-P<0.001 vs. UFH 300 U·kg^-1^ + Protamine 3.0 mg·kg^-1^; l-P<0.001 vs. UFH 900 U·kg^-1^, Mann-Whitney test. Results are shown as mean ± SD, n = 8–10.

Similarly to aPTT measurement, UFH prolonged the tail transection bleeding time. Dex40-GTMAC3 in the dose of 2.5 mg·kg^-1^ b.w., Dex6-GTMAC and GCD-GTMAC2 partially but significantly shortened bleeding time prolonged by 300 U·kg^-1^ b.w. of UFH (Fig. 6). When administered in the ratio of 2.5 mg for every 100 U·kg^-1^ b.w of UFH, Dex40-GTMAC3 completely reversed tail bleeding time prolonged by 300 or 900 U·kg^-1^ b.w of UFH to the control value (Fig. 6). Dex40-GTMAC3 administered alone did influence bleeding time (111.3 ± 18.9 sec.) in comparison to vehicle (104.3 ± 4.6 sec.).

### Reversal of plasma anti-Xa activity by the cationic polymers

**Fig 7:**
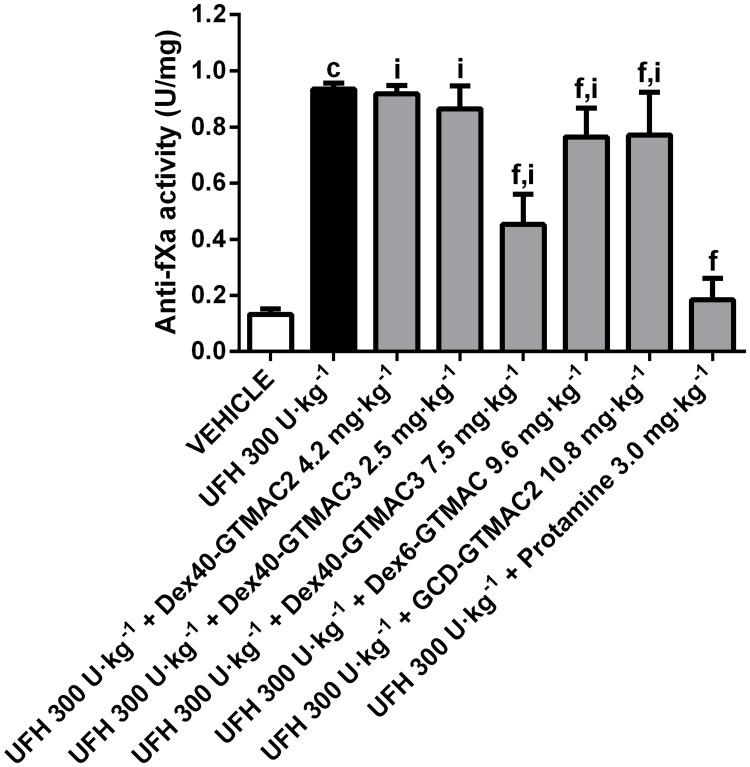
Reversal of plasma anti-fXa activity by the cationic polymers. Anti-fXa activity in Wistar rats treated with vehicle (PBS), UFH (300 U·kg^-1^) alone or followed by Dex40-GTMAC2 (4.2 mg·kg^-1^), Dex40-GTMAC3 (2.5 and 7.5 mg·kg^-1^), Dex6-GTMAC (9.6 mg·kg^-1^), GCD-GTMAC2 (10.8 mg·kg^-1^), and protamine (3.0 mg·kg^-1^), c-P<0.001 vs. vehicle; f-P<0.001 vs. UFH 300 U·kg^-1^; i-P<0.001 vs. UFH 300 U·kg^-1^ + Protamine 3.0 mg·kg^-1^, Mann-Whitney test. Results are shown as mean ± SD, n = 8–10.

Dex40-GTMAC3 and protamine significantly reversed increase of anti-Xa activity in UFH-treated animals (Fig. 7). Other polymers were completely or partially ineffective.

### Blood count

UFH significantly increased white blood cells (WBC). This increase of WBC was completely reversed to control value by protamine injection. All modified polymers partially, but significantly, inhibited increase of WBC in UFH treated animals. Only Dex40-GTMAC3 did not change any other blood count. GCD-GTMAC2 significantly changed red blood cell count, hemoglobin, and hematocrit values. All results are presented in Table 2.

**Table 2:** Body weights and complete blood count results after arterial thrombosis stimulation in rats, means ± SD.

	VEH	UFH300 U·kg^-1^	UFH300 U·kg^-1^+ Dex40-GTMAC24.2 mg·kg^-1^	UFH300 U·kg^-1^+ Dex40-GTMAC32.5 mg·kg^-1^	UFH300 U·kg^-1^+ Dex40-GTMAC37.5 mg·kg^-1^	UFH300 U·kg^-1^+ Dex6-GTMAC9.6 mg·kg^-1^	UFH300 U·kg^-1^+ GCD-GTMAC210.8 mg·kg^-1^	UFH300 U·kg^-1^+ Protamine 3.0 mg·kg^-1^
**N**	10	10	8	8	8	8	8	8
**Body weight (g)**	217.9 ± 9.4	216.8 ± 9.1	221.3 ± 12.4	215 ± 6.5	219.4 ± 14.0	216.0 ± 11.2	216.1 ± 10.9	219.5 ± 13.0
**WBC (10** ^**3**^ **/mm** ^**3**^ **)**	2.1 ± 0.2	3.7 ± 0.6 c	3.0 ± 0.7 c,d,h	2.6 ± 0.7 a,e	3.0 ± 0.4 c,d,g	3.0 ± 0.7 b,d,h	2.7 ± 0.8 d	2.0 ± 0.3 f
**RBC (10** ^**6**^ **/mm** ^**3**^ **)**	6.9 ± 0.2	6.8 ± 0.4	7.0 ± 0.6	6.4 ± 0.9	6.8 ± 0.3	6.5 ± 0.3 c	7.5 ± 0.3 c,f,i	6.6 ± 0.3 a
**HGB (g/dl)**	13.6 ± 0.4	13.7 ± 0.5	15.3 ± 0.8 c,f,i	13.8 ± 0.9	13.3 ± 0.6	13.6 ± 0.4 g	14.7 ± 0.9 b,e,h	13.1 ± 0.8 d
**HCT (%)**	40.5 ± 1.2	39.2 ± 2.3	41.7 ± 4.5	37.8 ± 5.5	39.8 ± 1.4	38.6 ± 1.3 b	43.7 ± 2.6 b,f,h	39.3 ± 1.7
**MCV (μm** ^**3**^ **)**	58.8 ± 0.6	59.0 ± 1.6	58.1 ± 2.5	58.9 ± 1.9	59.0 ± 1.8	59.4 ± 1.1	59.0 ± 1.1	59.7 ± 1.2
**MCH (pg)**	19.5 ± 0.3	20.5 ± 0.7 b	21.6 ± 1.8 b,g	21.9 ± 2.5 a,g	19.8 ± 0.8	21.0 ± 0.6 c,h	19.7 ± 0.6 d	19.8 ± 0.5 d
**MCHC (g/dl)**	33.3 ± 0.2	34.0 ± 0.5 c	37.2 ± 4.1 c,h	37.3 ± 4.5 b,h	33.6 ± 0.7	35.3 ± 0.6 c,f,i	33.6 ±0.8	33.2 ± 0.9 d
**PLT (10** ^**3**^ **/mm** ^**3**^ **)**	644 ± 23	636 ± 34	636 ± 97	662 ± 78	603 ± 104	657 ± 97	633 ± 36	651 ± 51

a-P<0.05,

b-P<0.01,

c-P<0.001 vs. vehicle;

d-P<0.05,

e-P<0.01,

f-P<0.001 vs. UFH 300 U·kg^-1^;

g-P<0.05,

h-P<0.01,

i-P<0.001 vs. UFH 300 U·kg^-1^ + Protamine 3.0 mg·kg^-1^, Mann-Whitney test.

WBC: white blood cells, RBC: red blood cells, HGB: hemoglobin, HCT: hematocrit, MCV: mean corpuscular volume, MCH: mean corpuscular hemoglobin, MCHC: mean corpuscular hemoglobin concentration, PLT: blood platelets. Results are shown as mean ± SD, n = 8–10.

### Effects of the cationic polymers on blood pressure in rats

**Fig 8:**
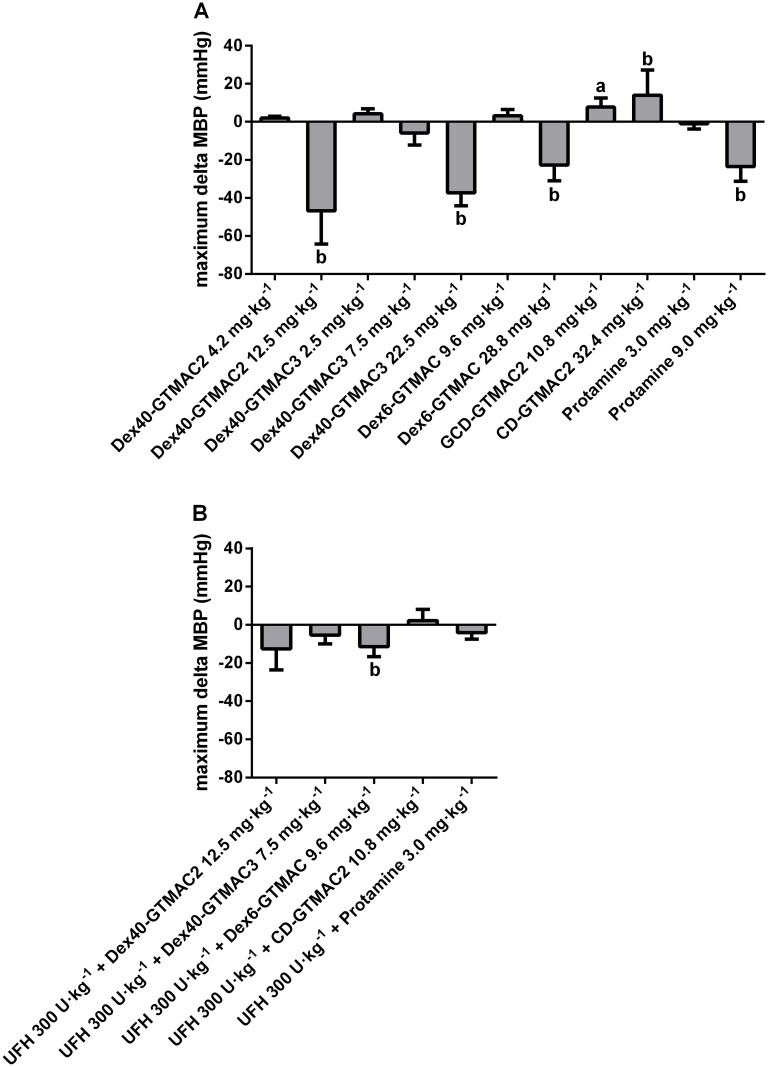
Effects of the cationic polymers on blood pressure in rats. MBP change registered 60 min after iv administration of Dex40-GTMAC2 (4.2 and 12.5 mg·kg^-1^), Dex40-GTMAC3 (2.5, 7.5, and 22.5 mg·kg^-1^), Dex6-GTMAC (9.6 and 28.8 mg·kg^-1^), GCD-GTMAC2 (10.8 and 32.4 mg·kg^-1^), and protamine (3.0 and 9.0 mg·kg^-1^) (A) and UFH (300 U·kg^-1^) followed by the injection of Dex40-GTMAC2 (12.5 mg·kg^-1^), Dex40-GTMAC3 (7.5 mg·kg^-1^), Dex6-GTMAC (9.6 mg·kg^-1^), GCD-GTMAC2 (10.8 mg·kg^-1^), and protamine (3.0 mg·kg^-1^) (B), a-P<0.05, b-P<0.01 vs. vehicle, Mann-Whitney test. Results are shown as mean ±SD, n = 4–6.

MBP courses in rats one hour after administration of the polymers and protamine are presented in Fig. A and B in S3 File. The maximum magnitude of MBP change is shown in Fig. 8A. MBP decreased in groups treated with modified Dex and protamine whereas it increased in animals treated with cationic GCD. In the group treated with Dex40-GTMAC2 MBP decreased by almost half in 15^th^ minute if the polymer was injected alone in the dose that efficiently reversed previous anticoagulant effects of UFH. We also found significant, but much weaker and temporary change of MBP shortly after administration of Dex40-GTMAC3 (decrease in MBP) and GCD-GTMAC2 (increase in MBP). Further increasing of the doses resulted in sustained significant drop in MBP in all animals including protamine treated group. If UFH was injected 3 minutes before the administration of the cationic polymers, only Dex40-GTMAC2 and Dex6-GTMAC decreased MBP by 13 and 11%, respectively, whereas Dex40-GTMAC3 and protamine did not (Fig. 8B). One polymer (Dex40-PAH-Arg) was extremely hypotensive, and three others (Dex40-Spm, Pul-GTMAC and HPC-APTMAC2) were lethal. Thus, we discontinued studying of these polymers. The results of experiment preformed in one rat are presented in Fig. C in S3 File.

### Evaluation of the immune response to the cationic polymers

**Fig 9:**
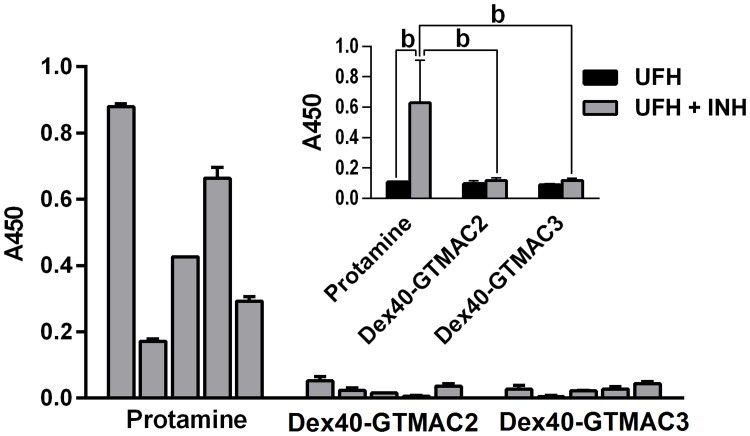
Evaluation of the immune response to the cationic polymers. Humoral immune response evaluation by ELISA on day 36 of the experiment. The levels of IgG specific toward different UFH inhibitors in individual mice presented as values of absorbance in ELISA test. The inserted graph shows mean ELISA signals corresponding to the levels of anti-protamine-, anti-Dex40-GTMAC2- or Dex40-GTMAC3-IgG present in sera of mice immunized with matching UFH-antidotes (gray bars) compared to the signals from sera of control mice treated with UFH alone (black bars). All sera applied in 1:100 dilution, b-P<0.01, Mann-Whitney test. Results are shown as mean ± SD, n = 5.

Since no control antibodies specific to protamine, Dex40-GTMAC2, and Dex40-GTMAC3 were available, antigen coating in ELISA was confirmed with spectroscopic and colorimetric methods. Both early (IgM-class) and mature (IgG-class) phases of humoral immune response were evaluated. No IgM antibodies specific to any of the antigens were detected. Given low affinity of emerging IgM-class immunoglobulins in early immune response, it is possible that weakly-binding unspecific background antibodies produced most of the ELISA signal. IgG-class response, however, was detectable in all mice in the protamine group beginning from day 21^st^ on. On day 36 the mean protamine-specific IgG signal in the protamine group was significantly higher than the background signal in the UFH group (P<0.01). In contrast to this result, no significant levels of IgG specific to Dex40-GTMAC2 and Dex40-GTMAC3 were detected (Fig. 9). To further evaluate an immunogenic potential of the antigens tested, we measured and weighed spleens of all animals in the immunization experiments since strong immunogens are known to induce splenomegaly. However, no significant differences in spleen size or weight were detected in any of the experimental groups.

## Discussion

The main goal of the study presented in this paper was to search for a new UFH antidote that would be safer than protamine, while having similar antiheparin potency. Among fourteen initially synthesized cationic derivatives of polysaccharides with various structures (both linear and cyclic), molecular weights (from 1 up to 200 kDa), and charge densities (degree of substitution with cationic groups ranging from 0.1 to more than 4), using *in vitro* and *in vivo* assays we found the most potent and the safest one.

Present work is a continuation of our previous studies on dextran and hydroxypropylcellulose, and included two new polysaccharides, i.e., pullulan and γ-cyclodextrin, while we discontinued experiments on chitosan-based polymer. We disqualified some of the obtained polysaccharides at a quite early stage of the studies. One polymer was poorly soluble (Dex40-PAH), while five others (Dex1-GTMAC, Dex40-GTMAC1, Dex40-APTMAC, GCD-GTMAC1, and HPC-APTMAC1) lacked *in vitro* UFH binding activity, the property we considered a prerequisite, probably due to low cationic charge density. The remaining eight polymers, all of which successfully passed *in vitro* tests, were qualified for further experiments. We found that four of them (Dex40-Spm, Dex40-PAH-Arg, Pul-GTMAC and HPC-APTMAC2) were lethal or extremely hypotensive at the preliminary stages of the *in vivo* study, thus experiments on those derivatives were discontinued to reduce the number of scarified animals according the 3R rule. Finally, we evaluated *in vivo* four polymers: Dex6-GTMAC, Dex40-GTMAC2, Dex40-GTMAC3, and GCD-GTMAC2. Among them the performance of Dex40-GTMAC2 was the poorest. When administered in non-hypotensive dose, it did not inhibit anticoagulatory and antithrombotic activity of UFH, although it lacked immunogenicity. All three remaining polymers significantly reversed action of UFH. We expected good efficacy in the case of Dex40-GTMAC3, as we had previously found partial antiheparin activity of a similar, but less cationic, 40 kDa dextran [17]. The results with GCD-GTAC2 were also not surprising, since others showed that a cationic polymer with a cyclic structure (PM102) successfully binds heparins [31] and a β-cyclodextrin-containing polycation neutralizes anionic aptamer-based anticoagulants [32]. However, the heparin reversal property of Dex6-GTMAC was unexpected and here we report for the first time strong antiheparin activity of a low molecular weight (6 kDa) cationic dextran.

Among these three promising polymers, we found that Dex40-GTMAC3 is the most potent and the safest antidote of UFH. Unlike Dex40-GTMAC3, Dex6-GTMAC decreased, and GCD-GTMAC increased red blood cell count, hematocrit and hemoglobin values. Dex6-GTMAC was hypotensive when administered in efficient antiheparin dose together with UFH. Similarly to others [32], we observed increase of MBP after intravenous injection of cationic β-cyclodextrin, alone or with UFH. In contrast, all hematological and hemodynamic values of animals receiving Dex40-GTMAC3 remained within error of their baseline level. Only Dex40-GTMAC3 and protamine comparably and fully restored the parameters of the thrombosis development, i.e., thrombus weight and bleeding time, and typical measures of heparins action, i.e., aPTT and anti-fXa activity, to control values. Moreover, Dex40-GTMAC3 did not elicit a detectable humoral immune response at doses that allow for complete neutralisation of UFH. The heparinisation/neutralisation regimen in BALB/c mice was repeated 5 times, once every week. This was aimed to mimic the clinical situation of dialysis patients, who are exposed to frequent heparin administration and reversal. In contrast, protamine—even if used at lower doses than Dex40-GTMAC3—was immunogenic in all animals and induced strong IgG responses. While this difference in immunogenic potential may be caused by the very chemical nature of the reversal agents (protein vs. polysaccharide), it can have significant clinical implications if the novel heparin reversal agents are to be used in medical practice.

According to summary of product characteristics, 300 U·kg^-1^ b.w. of UFH is a maximal single dose used during cardiopulmonary bypass, thus higher dose of UFH may be considered as overdose. In order to simulate emergency or overdosage of UFH we administered a dose of 3 x 300 U·kg^-1^ b.w. We found that Dex40-GTMAC3 again completely reversed antithrombotic and anticoagulant activity of UFH. It is known that protamine administered in doses exceeding the ratio of 1 mg per 100 U of UFH may exert anticoagulant activity [30]. We found that Dex40-GTMAC3 administered alone to rat in dose of 7.5 mg·kg^-1^ b.w. clinically insignificantly prolonged aPTT, without changing of thrombus weight, bleeding time and blood pressure. However, the results of in vitro aPTT assay (2 x aPTT prolongation at concentration of 100 mg·l^-1^) and the blood pressure measurement (37 mmHg decrease caused by Dex40-GTMAC3 administered in dose of 22.5 mg·kg^-1^ b.w.) indicated that, similarly to protamine, significant exceeding the ratio of 2.5 mg·kg^-1^ b.w. of Dex40-GTMAC3 per 100 U of UFH could not be well tolerated by patients. It seems that positively charged compounds at high concentrations (higher than therapeutic) exert antithrombotic activity themselves, and the mechanism could be associated with their hypotensive activity. Nevertheless, in contrast to delparantag—a novel, very promising reversal agent of UFH/low molecular weight heparin/pentasaccharide, in case of which the enrolment to clinical trial was stopped because of significant hypotension [9], Dex40-GTMAC3 is normotensive in therapeutic doses.

The interspecies differences between rodents and human can often be a reason of drug discovery failure. They are even more important in the evaluation of agents used in the treatment of complex haemostasis and hemodynamic disorders. To make the data on the inhibition of antithrombotic activity of UFH more meaningful we also examined the efficacy of the Dex40-GTMAC3 in mice using a model of thrombosis in which the lesion was produced inside abdominal vein, simulating contact of blood with thrombogenic surface [27]. Moreover, in this model the time of observation was extended to 6 hours to be closer to long open heart surgeries.

In contrast to many studies focusing on heparin-binding properties of protamine alternatives, for example lactoferrin [33], methylene blue, vancomycin, hexadimethrine bromide [34], or chemically-modified inactive antithrombin [35], Dex40-GTMAC3 efficacy was confirmed *in vitro* and *in vivo* in both mice and rats. Nevertheless, we are aware that further primate studies and clinical trial are required to confirm efficacy and safety in humans. Several UFH antidotes that had been promising *in vitro* failed at the later stages of development. For example, the recombinant human fVIIa given to patients increased the incidence of venous thromboembolism, pulmonary embolism, and myocardial infarction [36]. Platelet factor 4 as heparin antidote did not complete clinical trial, probably due to safety reasons (it increases the risk of heparin-induced thrombocytopenia) [37,38].

In agreement with other reports [39], we found that protamine is immunogenic, whereas Dex40-GTMAC3 is not. This is an important advantage of Dex40-GTMAC3 over the protamine and alternative UHF reversal agents. Other protein-based antidotes, such as heparinase I [40], lactoferrin [33], or low molecular weight protamine [41,42] may induce immunogenic responses similar to protamine. Moreover, availability of Dex40-GTMAC3 does not depend on natural fish sources, which is the case of protamine sulfate. Lack of detectable immune response towards Dex40-GTMAC3 indicates that, in contrast to protamine, it could be safely administered to patients with fish allergy and to diabetics treated with protamine insulin.

It was suggested that some complications of cardiopulmonary bypass, such as bleeding or thrombotic risk, thrombocytopenia, or neurological disorders [43–46] could be related to administration of high doses of protamine [47]. However, it is very hard to separate effects of protamine treatment from those related to the use of cardiopulmonary bypass in clinical studies. Our experimental study to some extent supplements incomplete preclinical safety profile of protamine, pointing on its hypotensive and immunogenic properties.

Many polymers in our study were excluded during *in vitro* screening phase or after injection to just a few animals due to their toxicity. We showed here these negative data, because they may be valuable for others to exclude these compounds from their *in vivo* studies, even in different areas of application. For example, cationic polymers have been applied as gene carriers in nonviral gene therapy [48] and as drug delivery systems [49]; their potential use as anticancer drugs has also been suggested [50]. Moreover, a hydrogel containing GTMAC-modified polysaccharide can be applied to cell encapsulation and protein delivery [51,52]. Based on our results we can claim that high molecular weight linear polymers perform better as therapeutics than the cyclic or low molecular weight cationic polysaccharides.

We are aware that approving a first-in-man study will require not only pharmacodynamics and general safety data, but also pharmacokinetics, organ distribution and toxicity studies. The results may exclude Dex40-GTMAC3 from further development, thus we keep searching for agents in different polymer classes. We have recently shown that polyallylamine containing arginine moieties (PAH-ARG) could be a promising agent neutralizing protamine [23].

In conclusion, our team developed a novel, potent and safe heparin antidote—Dex40-GTMAC3, a 40 kDa dextran substituted with 0.65 GTMAC groups per a glucose unit on the average. Basing on our non-clinical evaluation of Dex40-GTMAC3 and taking into account the preclinical registration data and clinical safety literature of protamine, we believe that the novel polymer has very promising properties and can potentially substitute protamine as the UFH antidote.

## Supporting Information

S1 File Synthesis of polymers. (PDF)

S2 File Characteristics of the polymers. (PDF)

S3 File Mean blood pressure course in rats one hour after administration of the polymers and protamine. (PDF)
