# Supplementary material for: Nonclinical Evaluation of Novel Cationically Modified Polysaccharide Antidotes for Unfractionated Heparin
Source: PLoS One. 2015 Mar 17;10(3):e0119486. doi: 10.1371/journal.pone.0119486 (PMC4362941; doi:10.1371/journal.pone.0119486)
Supplement: S1 File — (PDF) [file pone.0119486.s001.pdf]

S1 File. Synthesis of polymers.

Synthesis of polysaccharides substituted with glycidyltrimethylammonium chloride (GTMAC)

The derivatives of dextran (Dex), pullulan (Pul), and γ-cyclodextrin (GCD) substituted with GTMAC were synthesized using the general procedure described previously [1]. Two grams of a polysaccharide were dissolved in 100 ml of distilled water. Then, NaOH was added and the solution was stirred with a magnetic stirrer and heated to 60°C. In the next step GTMAC was added and the mixture was heated and kept at 60°C while being stirred. The exact amounts of sodium base and GTMAC, and the reaction times are given in Table A. Then, the reaction mixtures were cooled and the reaction products (except for the reaction of Dex1-GTMAC and GCD) were transferred to the dialysis tubes with the Mw cutoff value of 12.8 kDa (in the case of Dex6-GTMAC the dialysis tube with the Mw cutoff value 3kDa was used). The dialysis was carried out against distilled water until the conductivity of the solution outside the tube decreased down to 2 μS. The modified polymers were then isolated from the solution using the freeze-drying technique. In the case of the derivatives of Dex1-GTMAC and GCD the reaction solution was neutralized with HCl to pH ~7 and then the product was precipitated in cold acetone. The precipitate was rinsed with acetone twice. The resulting product was again washed with methanol (Dex1-GTMAC) or ethanol (GCD derivatives) to completely wash out the sodium chloride and unreacted GTMAC. Purification of the products was confirmed with measurements of the conductivity of the washing liquid. The obtained Dex1-GTMAC and GCD derivatives were dried in a vacuum oven for 24h.

Table A. The synthesis conditions for the polysaccharides modified with GMTAC.

Polymer	Substrate polysaccharide	Weight of NaOH (mg)	Volume of GTMAC (ml)	Reaction time (h)
**Dex1-GTMAC**	Dex, 1 kDa	400 mg	24 ml	4
**Dex6-GTMAC**	Dex, 6 kDa	400 mg	24 ml	4
**Dex40-GTMAC1**	Dex, 40 kDa	100 mg	12 ml	4
**Dex40-GTMAC2**	Dex, 40 kDa	400 mg	12 ml	4
**Dex40-GTMAC3**	Dex, 40 kDa	400 mg	24 ml	4
**Pul-GTMAC**	Pul	400 mg	24 ml	4
**GCD-GTMAC1**	GCD	400 mg	12 ml	2
**GCD-GTMAC2**	GCD	400 mg	24 ml	2

Synthesis of dextran (Dex) and hydroxypropylcellulose (HPC) substituted with N-acrylamidopropyl-N,N,N-trimethylammonium chloride (APTMAC)

The derivatives of dextran (Dex, 40 kDa) and hydroxypropylcellulose (HPC) substituted with APTMAC were synthesized using the general procedure described previously [1]. The experimental conditions of the synthesis are given in Table B. In a three-necked flask 1.5 g of the respective polysaccharide was dissolved in 15 mL of a solvent. The solution was degassed by bubbling with nitrogen for 30 min and a solution of the initiator was added. After 5 min 75 wt% solution of APTMAC in water was added. The reaction mixture was heated at 70 ^{o}C for 3 h under constant mixing with a magnetic stirrer and under bubbling with nitrogen. Then, the mixture was cooled and dialyzed first against the solvent used and after 12 h against a mixture of the solvent and water. The fraction of water had been gradually increased for a week, and finally the dialysis was performed in pure water. The dialysis was carried out against water for 2 more weeks. The polymers obtained (Dex40-APTMAC and HPC-APTMAC) were isolated from the solution using the freeze-drying technique.

Table B. The synthesis conditions for the polysaccharides modified with APTMAC

Polymer	Modified polysaccharide	Solvent	Initiator solution	Weight of APTMAC solution
**Dex40-APTMAC**	Dex, 40 kDa	DMSO	1.5 g BPO in 15 ml DMSO	4.43 g
**HPC-APTMAC1**	HPC, 80 kDa	DMF	1.5 g BPO in 15 ml DMF	1.77 g in 16 ml DMF
**HPC-APTMAC2**	HPC 80 kDa	DMF	1.5 g BPO in 15 ml DMF	17.72 g

Synthesis of dextran (Dex) grafted with poly(allylamine hydrochloride) (PAH)

Allylamine hydrochloride was prepared by dropwise adding 4 g of allylamine to 7 ml of concentrated hydrochloric acid. Then, 1.5 g of Dex (40 kDa) was dissolved in 15 ml water. The Dex solution was degassed by bubbling with nitrogen for 30 min and a solution of the initiator (1.35 g of AAPH in 7.5 mL of degassed water) was added. After 5 min allylamine hydrochloride was added. The reaction mixture was heated at 55°C for 3 h under constant mixing with a magnetic stirrer and bubbled with nitrogen. The mixture was then cooled and dialyzed against water in the dialysis tubes (Mw cutoff value of 12 kDa). The obtained product (Dex40-PAH) was isolated from the solution using the freeze-drying technique.

Synthesis of dextran (Dex) grafted with poly(allylamine hydrochloride) (PAH) substituted with arginine (Arg)

The synthesis of dextran (Dex) grafted with poly(allylamine hydrochloride) (PAH) substituted with L-arginine (Arg) followed that described previously [2]. To the solution of 0.4 g Dex40-PAH in 10 ml of distilled water 120 mg of EDC, 60 mg of NHS, and 2 g of Arg were added. The mixture was heated to

60°C and stirred for 24 h. The mixture was then cooled down to room temperature and dialyzed against water for 7 days in a dialysis tube (Mw cutoff value of 12 kDa). Then, the obtained polymer (Dex40-PAH-Arg) was isolated from the aqueous solution by freeze-drying.

Synthesis of dextran substituted with spermine (Spm)

The synthesis of dextran (Dex) grafted with spermine (Spm) generally followed that described previously [3]. 0.5 g of Dex (40 kDa) was dissolved in 10 ml of DMSO followed by the addition of 1 g of CDI and 1 g of Spm dissolved in10 ml of DMSO. The reaction mixture was heated at 40°C for 2 h under constant mixing with a magnetic stirrer. Then, the mixture was cooled down and dialyzed in the dialysis tubes (Mw cutoff value of 12 kDa) against DMSO for 12 h against a mixture of DMSO and water. The fraction of water had been gradually increased for a week, and finally the dialysis had been performed against pure water for a week. The insoluble fraction was removed by filtration and the resulting polymer (Dex40-Spm) was isolated from the aqueous solution by freeze-drying.

Solubility of the polymers at physiological pH

Prior to the tests assessing the ability of the synthesized polymers to interact with heparin their solubility in PBS buffer (pH=7.4) was examined by mixing of 1 mg of the test substance and 1 ml of pH 7.4 PBS buffer. The solubility of the compound was checked visually and with a DLS measurement. All the materials, except for Dex40-PAH, were soluble at this concentration and in the DLS measurements no objects larger than 50 nm were found. Therefore, Dex40-PAH was excluded from UFH binding test and biological studies.

References

1. Kaminski K, Plonka M, Ciejka J, Szczubialka K, Nowakowska M, Lorkowska B, et al. Cationic derivatives of dextran and hydroxypropylcellulose as novel potential heparin antagonists. J Med Chem. 2011;54: 6586-6596.
2. Kamiński K, Kałaska B, Koczurkiewicz P, Michalik M, Szczubiałka K, Mogielnicki A, et al. New arginine substituted derivative of poly(allylamine hydrochloride) for heparin reversal. MedChemComm. 2014;5: 489-495.
3. Seki T, Fukushi N, Chono S, Morimoto K. Effects of sperminated polymers on the pulmonary absorption of insulin. J Control Rel. 2008;125: 246-251.
