# Supplementary material for: Nonclinical Evaluation of Novel Cationically Modified Polysaccharide Antidotes for Unfractionated Heparin
Source: PLoS One. 2015 Mar 17;10(3):e0119486. doi: 10.1371/journal.pone.0119486 (PMC4362941; doi:10.1371/journal.pone.0119486)
Supplement: S2 File — (PDF) [file pone.0119486.s002.pdf]

S2 File. Characteristics of the polymers.

GTMAC-substituted polysaccharides

Eight polymers were synthesized by substitution of the hydrogen atom of the hydroxyl groups with GTMAC. Their structures were confirmed using ^{1}H NMR (Figures A and B). A new signal at 3.1 ppm was found in the spectra of the products which was ascribed to the protons of the methyl groups of GTMAC. In the FT-IR spectra of all GTMAC-substituted polymers a weak band at 1480 cm^{-1} appeared that could be attributed to an asymmetric angular binding of the methyl groups of GTMAC (Figure C). The elemental analysis (Table A) also confirmed the attachment of GTMAC groups to the polysaccharide backbone and allowed calculation of the degree of substitution.

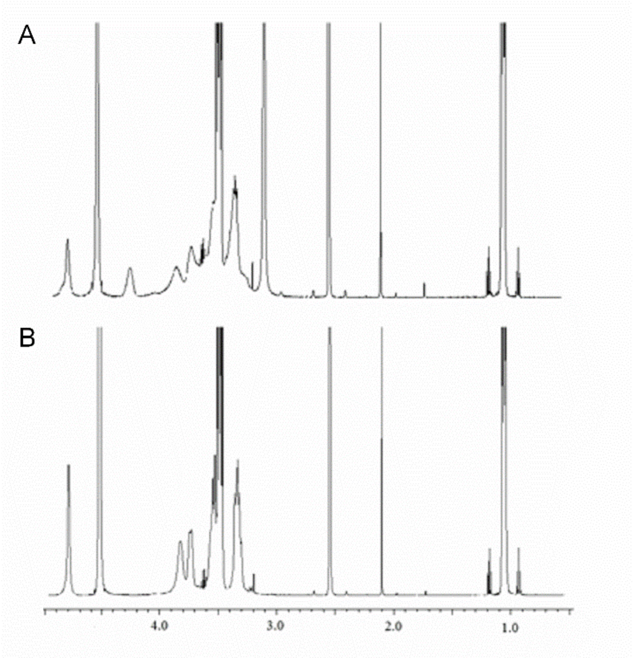

Figure A. ^{1}H NMR spectra of Dex40-GTMAC3 (A) and Dex40 (B). Signals: 4.8 ppm – water, 2.5 ppm – DMSO, the signals in the 3–4 ppm region originate from the hydrogens of Dex40 and Dex40-GTMAC3 attached to C-2, C-3, C-4, C-5 and C-6 carbon atoms and the hemiacetal C-1 resonance of these polymers occurs in the 4–5 ppm region. New signals were found in the spectrum of Dex40-GTMAC3 compared to Dex40 spectrum, i.e., a signal at 3.1 ppm which was ascribed to the methyl group protons of GTMAC and a signal at 4.2 ppm which was ascribed to the proton of the CH group closest to OH group in GTMAC.

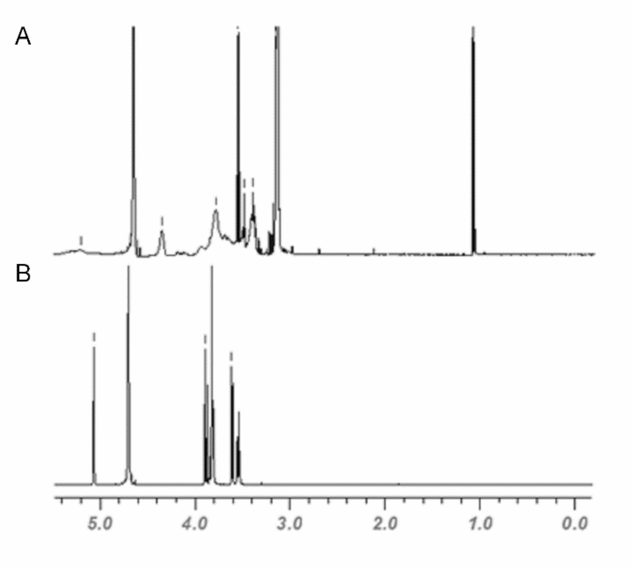

Figure B. ^{1}H NMR spectra of GCD-GTMAC2 (A) and GCD (B). Signals: 4.8 ppm – water, the signals in the 3–4 ppm region originate from the hydrogens of GCD and GCD-GTMAC2 attached to C-2, C-3, C-4, C-5 and C-6 carbon atoms. New signals were found in the spectrum of GCD-GTMAC2 compared to GCD spectrum, i.e., a signal at 3.1 ppm which was ascribed to the methyl group protons of GTMAC and a signal at 4.2 ppm which was ascribed to the proton of the CH group closest to OH group in GTMAC.

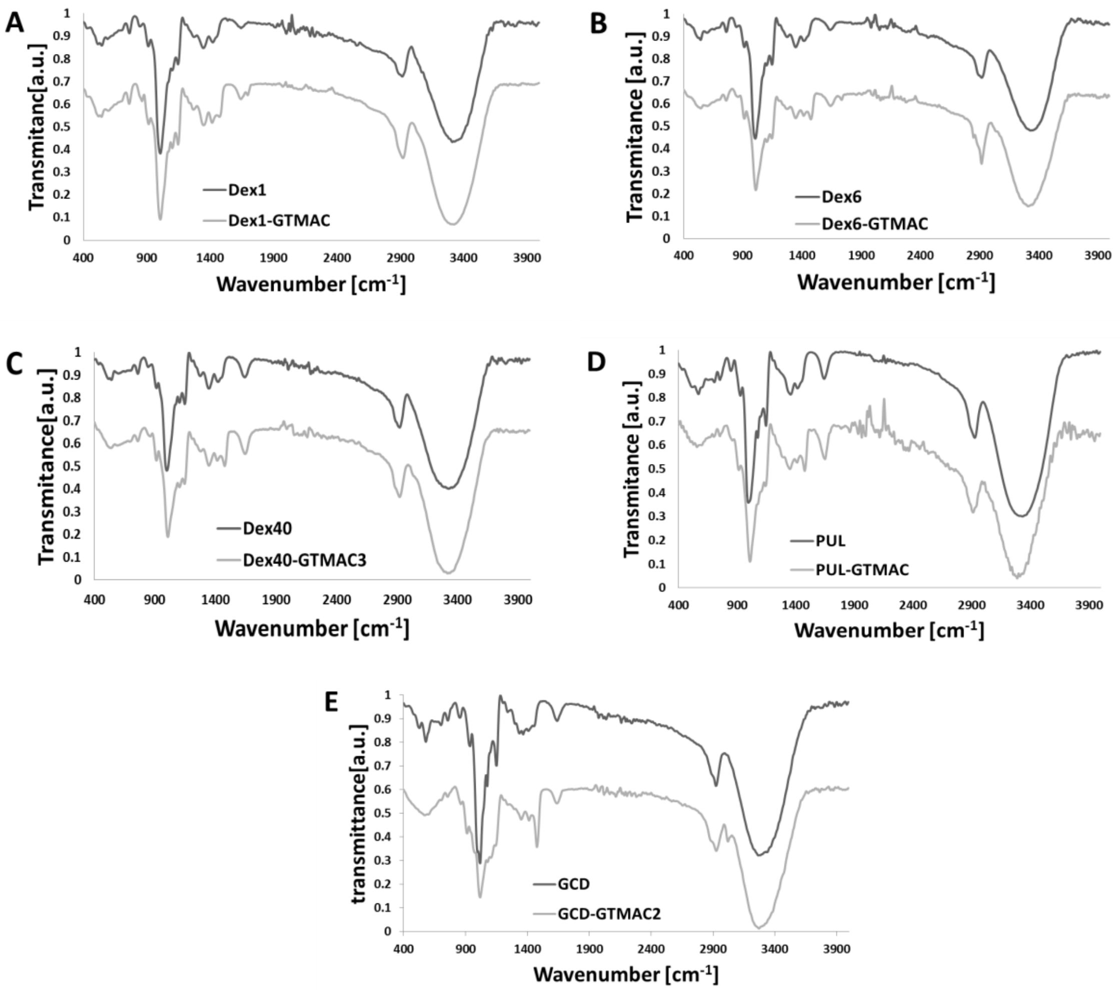

Figure C. FT-IR spectra of Dex1-GTMAC (A), Dex6-GTMAC (B), Dex40-GTMAC3 (C), Pul-GTMAC (D) and GCD-GTMAC2 (E).

Table A. Elemental composition

Polymer	Elemental composition (%)		
	C	H	N
**HPC-APTMAC1**	52,66	8,50	1,58
**HPC-APTMAC2**	50,33	9,07	9,29
**Dex40-GTMAC1**	40,60	6,72	0,92
**Dex40-GTMAC2**	41,37	7,07	2,03
**Dex40-GTMAC3**	42,28	7,35	2,66
**Dex40-APTMAC**	38,96	7,15	1,24
**Dex40-Spm**	45,94	7,83	9,62
**Dex40-PAH**	39,49	7,58	5,67
**Dex40-PAH-Arg**	43,25	7,24	3,95
**Dex6-GTMAC**	41,86	7,43	2,42
**Dex1-GTMAC**	41,20	7,28	1,83
**GCD-GTMAC1**	41,95	7,85	3,24
**GCD-GTMAC2**	40,05	7,94	3,89
**Pul-GTMAC**	43,30	7,47	3,00

APTMAC-grafted polysaccharides

Dex and HPC were grafted with APTMAC using the radical polymerization process. Three APTMAC-grafted polymers were obtained. The changes in the ^{1}H NMR and FT-IR spectra of HPC and Dex upon grafting were similar to those seen for GTMAC-substituted polymers, since both APTMAC and GTMAC contain methyl groups attached to the quaternary amine nitrogen. In the ^{1}H NMR spectrum of the products a signal at 3.1 ppm appeared coming from the methyl groups of APTMAC and a peak at 2.15 ppm appeared coming from the amide proton (Figures D and E). Moreover, a band at 1540 cm^{-1} corresponding to deformation vibrations of the -NH bond of the amide group appeared in the spectrum of APTMAC derivatives (Figure F). Using GPC it was verified that one peak was obtained in the chromatogram and its retention times in chromatograms obtained using two different detectors (UV-vis and IR) were identical; therefore, the formation of APTMAC homopolymer could be excluded (Figure G). Degree of substitution was calculated based on the elemental analysis results (Table A).

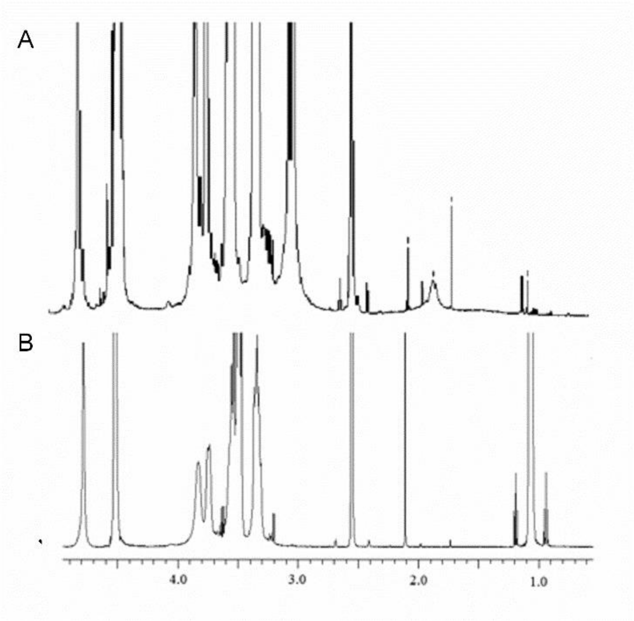

Figure D. ^{1}H NMR spectra of Dex40-APTMAC (A) and Dex40 (B). Signals: 4.8 ppm – water, 2.5 ppm - DMSO, the signals in the 3–4 ppm region originate from the hydrogens of Dex40 and Dex40-APTMAC attached to C-2, C-3, C-4, C-5 and C-6 carbon atoms and the hemiacetal C-1 resonance of these polymers occurs in the 4–5 ppm region. New signals was found in the spectrum of Dex40-APTMAC compared to Dex40 spectrum, i.e., a signal at 3.1 ppm which was ascribed to the methyl group protons of APTMAC and a signal at 2.15 ppm ascribed the amide proton.

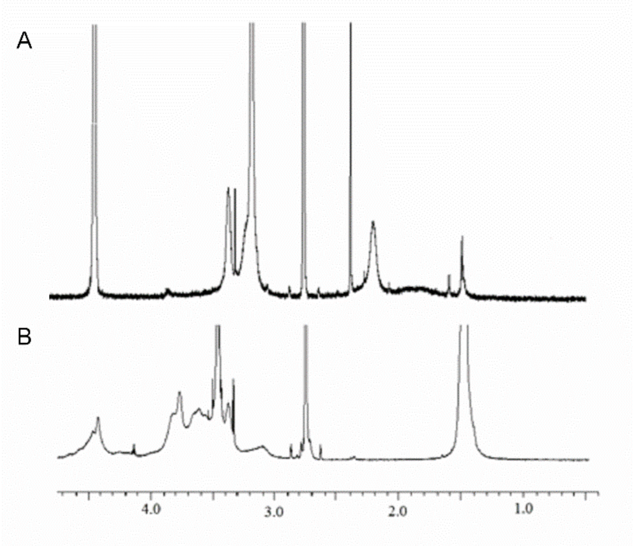

Figure E. ^{1}H NMR spectra of HPC-APTMAC2 (A) and HPC (B). Signals: 4.8 ppm – water, 2.5 ppm – DMSO, the signals in the 3–4 ppm region originate from the hydrogens of HPC and HPC-APTMAC2 attached to C-2, C-3, C-4, C-5 and C-6 carbon atoms the hemiacetal C-1 resonance of these polymers occurs in the 4–5 ppm region. New signals were found in the spectrum of HPC-APTMAC2 compared to HPC spectrum, i.e., a signal at 3.1 ppm which was ascribed to the methyl group protons of APTMAC and a signal at 2.15 ppm ascribed to the amide proton.

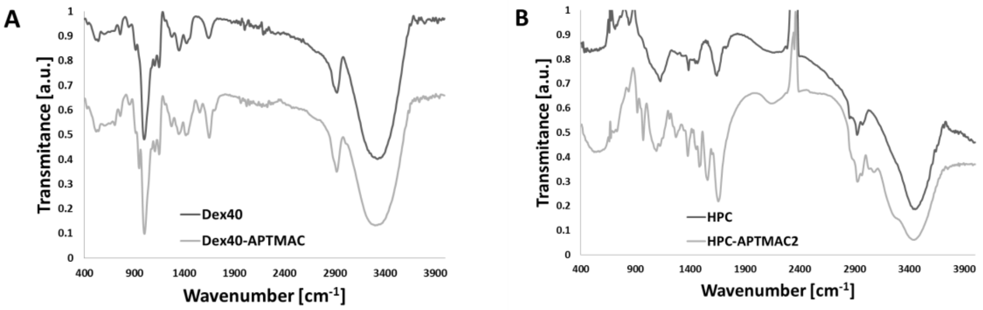

Figure F. FT-IR spectra of Dex40-APTMAC (A) and HPC-APTMAC2 (B).

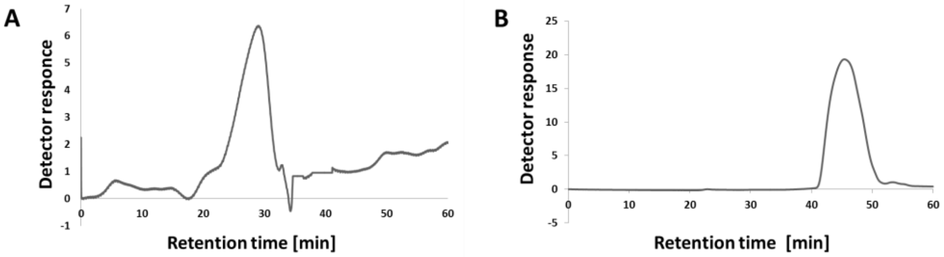

Figure G. GPC peaks of Dex40-APTMAC (A) and HPC-APTMAC2 (B).

PAH-grafted polysaccharides

Dex was cationically modified by grafting PAH using the radical polymerization process. The amino groups in the resulting graft polymer (Dex40-PAH) were then substituted with arginine to obtain Dex40-PAH-Arg polymer possessing two types of cationic groups. The structures of the cationic polysaccharides synthesized were confirmed using ^{1}H NMR and FT-IR spectroscopy. In the FT-IR spectra of PAH-grafted Dex derivatives characteristic bands are present confirming the presence of PAH chains grafted from the polysaccharide backbone, i.e. a band at 1533 cm^{-1} (NH_2 deformational vibration), 3200 cm^{-1} (NH, stretching) and 2800 cm^{-1} (NH, stretching) (Figure H). In ^{1}H NMR spectra a new signal at 1.8 ppm coming from CH_2 protons adjacent to NH_2 group appeared (Figure I). In the case of the FT-IR spectrum of Dex-PAH-Arg the new bands were observed compared to the Dex40-PAH spectrum, i.e. the bands at 1314 cm^{-1} and 1550 cm^{-1} originating from the symmetric deformation vibrations of NH_3^+ characteristic of amino acids (Figure H). In the ^{1}H NMR spectra the signals between 2.8 and 3.1 ppm originating from the CH_2 protons located close to the amino and amide groups, characteristic of the amino acid side chain groups, were observed (Figure I). The number of PAH and Arg units per one glucose unit according to elemental analysis were 1.17 (Dex40-PAH) and 0.10 (Dex40-PAH-Arg) (Table A).

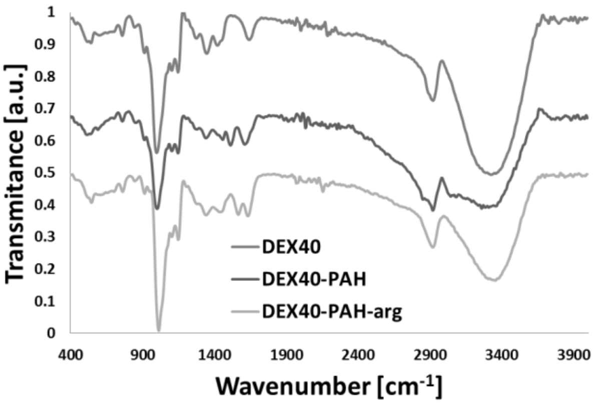

Figure H. FT-IR spectra of Dex40, Dex40-PAH, and Dex40-PAH-Arg.

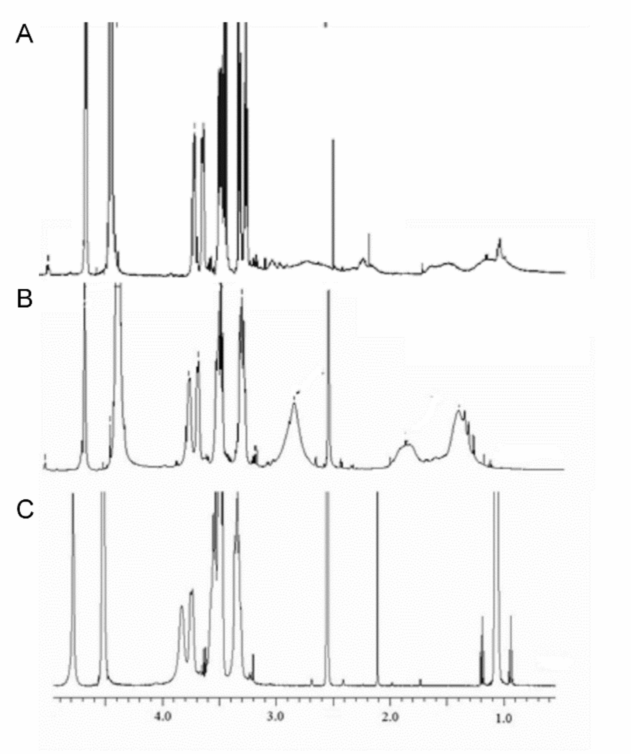

Figure I. ^{1}H NMR spectra of Dex40-PAH-Arg (A), Dex40-PAH (B), and Dex40 (C). Signals: 4.8 ppm – water, 2.5 ppm – DMSO, the signals in the 3–4 ppm region originate from the hydrogens of Dex40, Dex40-PAH and Dex40-PAH-Arg attached to C-2, C-3, C-4, C-5 and C-6 carbon atoms and the hemiacetal C-1 resonance of these polymers occurs in the 4–5 ppm region.. For Dex40-PAH a new signal was found in the spectrum at 1.8 ppm coming from the CH_2 protons adjacent to the NH_2 group. For Dex40-PAH-Arg the signals between 2.8 and 3.1 ppm were found originating from the CH_2 protons located close to the amino and amide groups, characteristic of the amino acid side chain groups.

Dextran substituted with spermine

The structure of Dex (Mw 40 kDa) substituted with spermine (Dex40-Spm) was confirmed using FT-IR spectroscopy. The new bands at 2882 cm^{-1} (NH, stretching), 1545 cm^{-1} (deformation vibrations of primary amine), 1279 cm^{-1} (C-N, stretching) are present in the spectrum of Dex40-Spm confirming the attachment of Spm to Dex (Figure J).

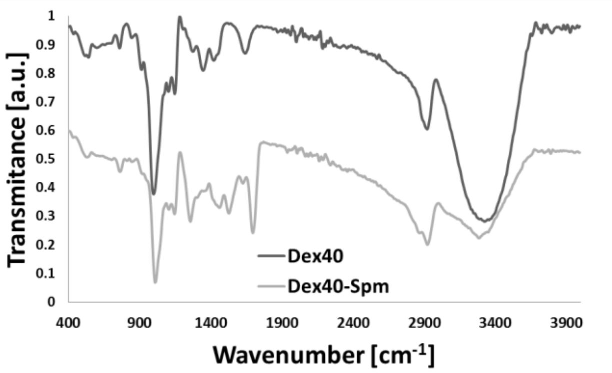

Figure J. FT-IR spectra of Dex40 and Dex40-Spm.
