# Supplementary material for: Nonclinical Evaluation of Novel Cationically Modified Polysaccharide Antidotes for Unfractionated Heparin
Source: PLoS One. 2015 Mar 17;10(3):e0119486. doi: 10.1371/journal.pone.0119486 (PMC4362941; doi:10.1371/journal.pone.0119486)
Supplement: S3 File — (PDF) [file pone.0119486.s003.pdf]

S3 File. Mean blood pressure (MBP) course in rats one hour after administration of the polymers and protamine.

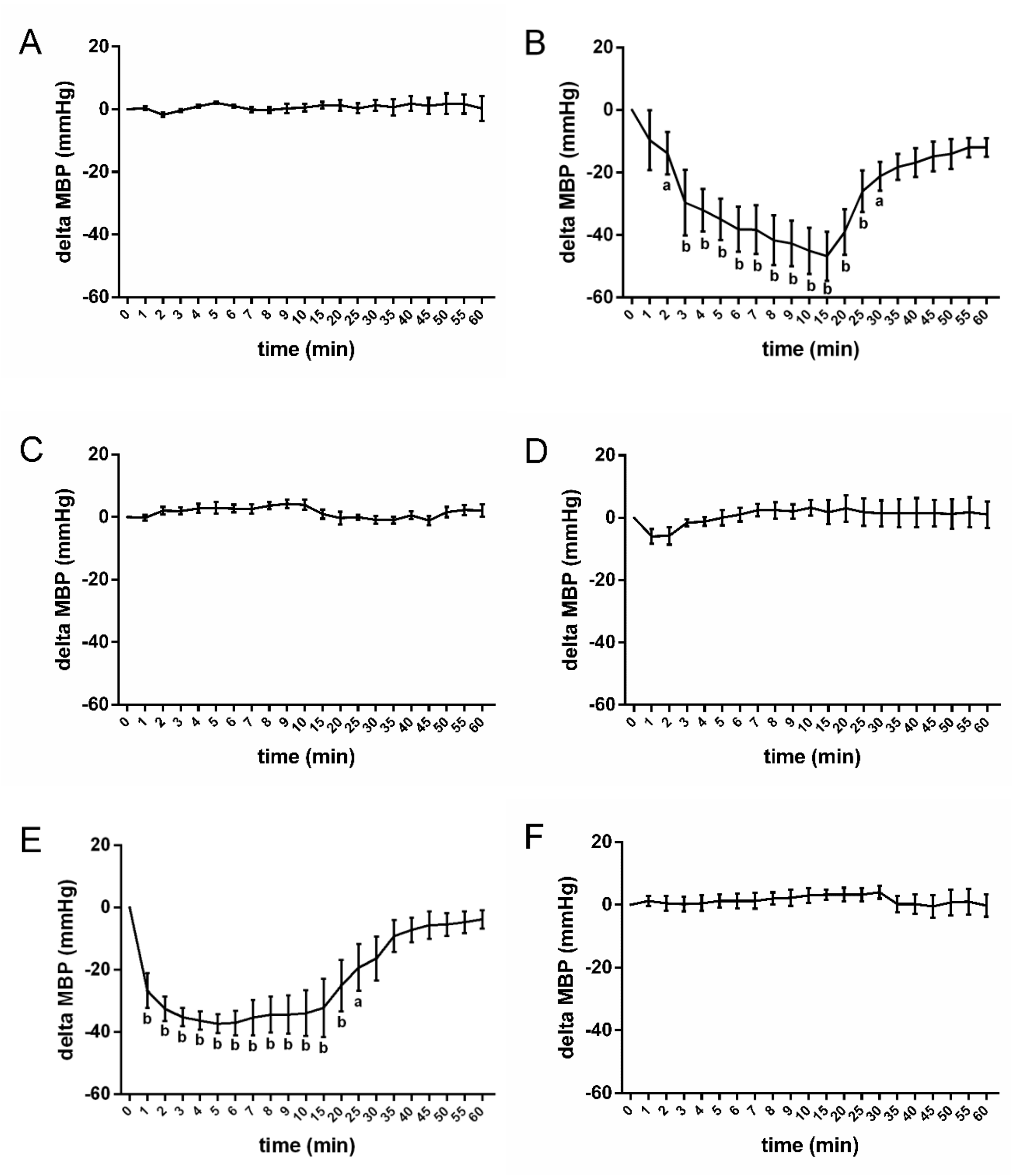

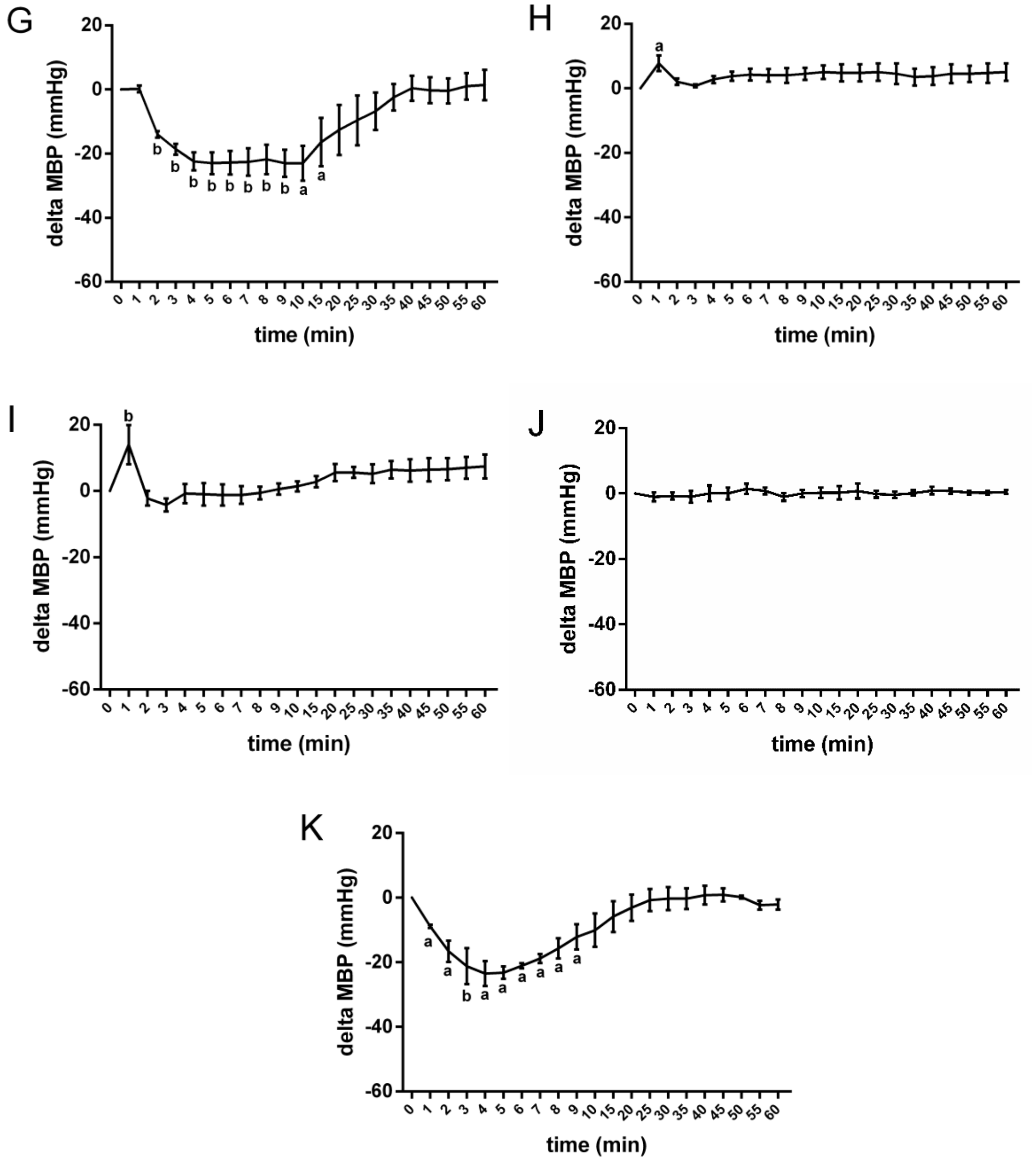

Figure A. Lines represent the course of MBP registered for 60 min after *iv* administration of Dex40-GTMAC2 (4.2 and 12.5 mg·kg^{-1}; A and B, respectively), Dex40-GTMAC3 (2.5, 7.5 and 22.5 mg·kg^{-1}; C, D and E, respectively), Dex6-GTMAC (9.6 and 28.8 mg·kg^{-1}; F and G, respectively), GCD-GTMAC2 (10.8 and 32.4 mg·kg^{-1}; H and I, respectively), and protamine (3 and 9 mg·kg^{-1}; J and K, respectively), a-$P<0.05$, b-$P<0.01$ vs. vehicle, Mann-Whitney test. Results are shown as mean ± SEM, n = 4-5.

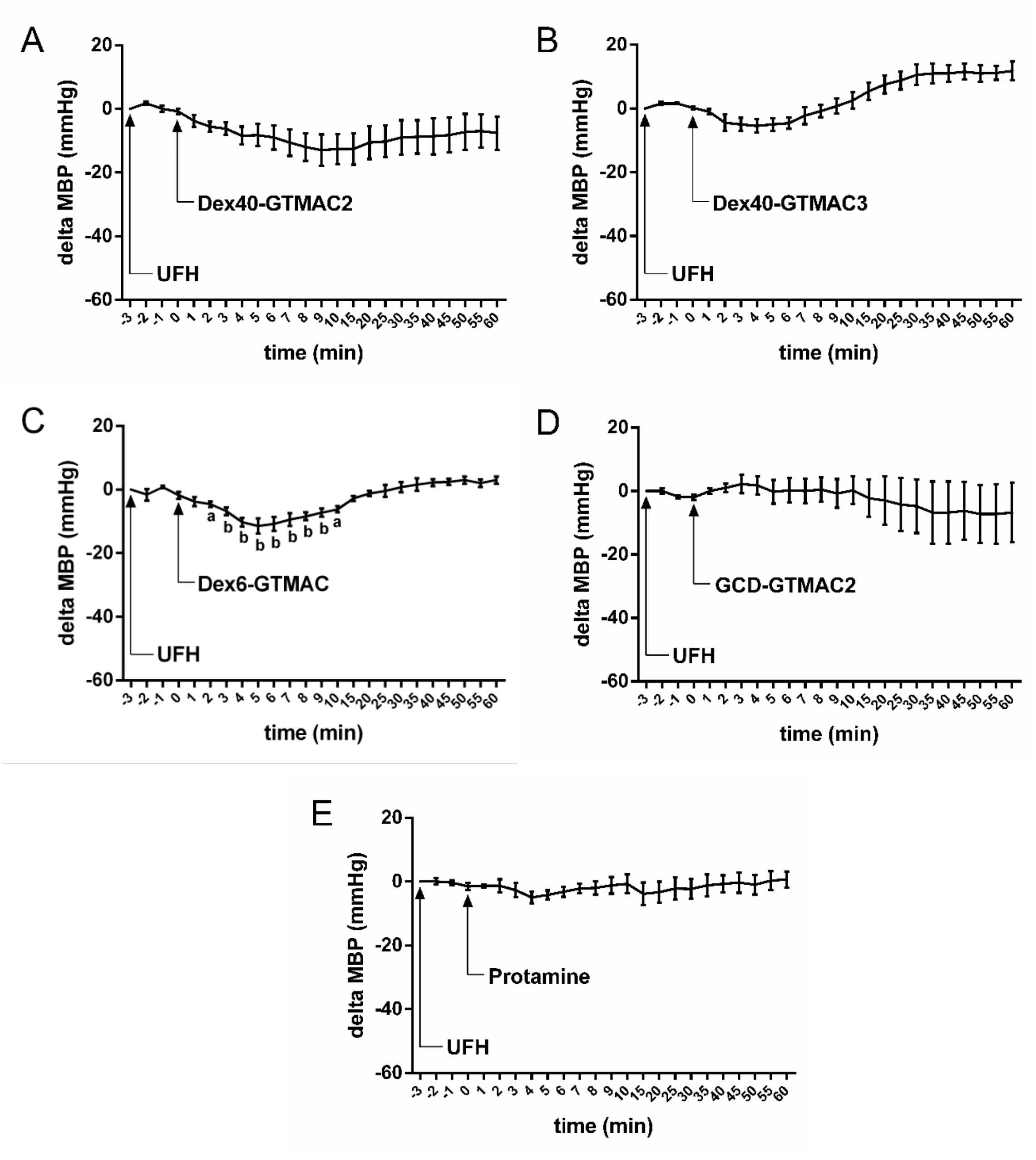

Figure B. Lines represent course of mean blood pressure (MBP) registered for 60 min after *iv* administration of UFH (300 U·kg^{-1}) followed by Dex40-GTMAC2 (12.5 mg·kg^{-1}; A), Dex40-GTMAC3 (7.5 mg·kg^{-1}; B), Dex6-GTMAC (9.6 mg·kg^{-1}; C), GCD-GTMAC2 (10.8 mg·kg^{-1}; D), and protamine (3 mg·kg^{-1}; E), a-P<0.05, b-P<0.01 vs. vehicle, Mann-Whitney test. Results are shown as mean ± SEM, n = 4-6.

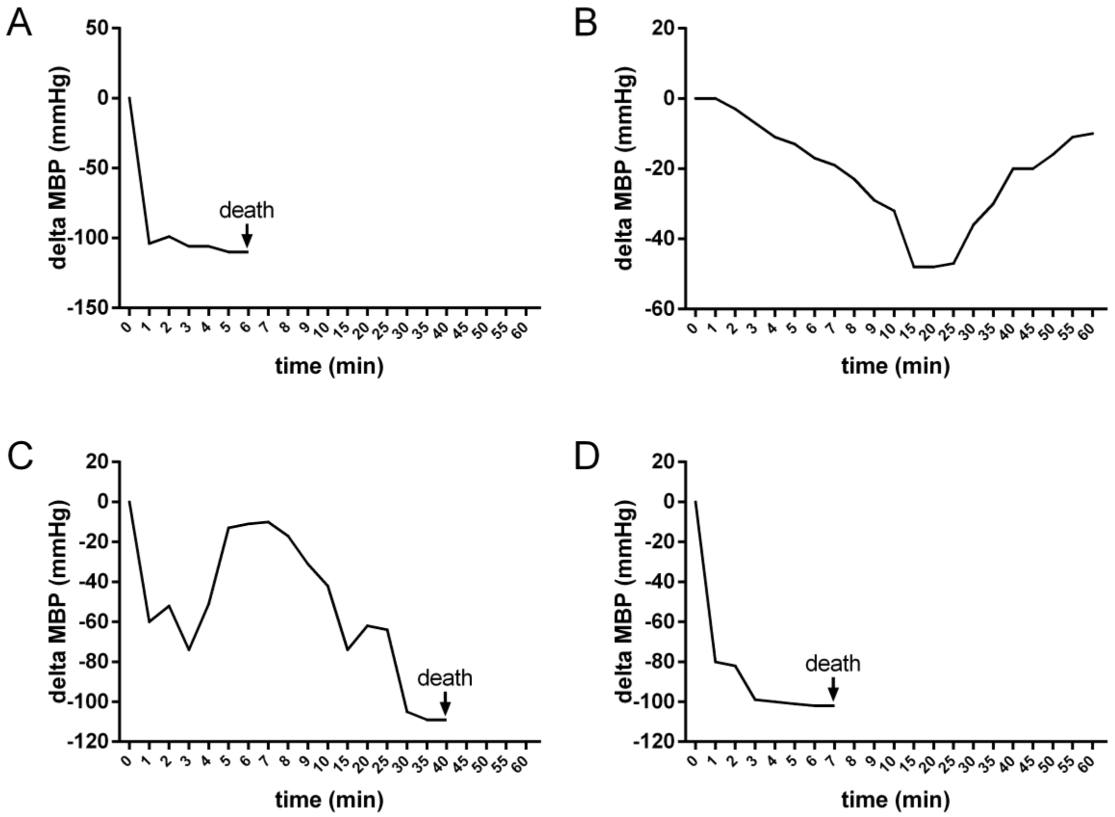

Figure C. Lines represent the course of MBP registered for 60 min after *iv* administration of Dex40-Spm (9 mg·kg^{-1}; A), Dex40-PAH-Arg (32.4 mg·kg^{-1}; B), Pul-GTMAC (21.6 mg·kg^{-1}; C), and HPC-APTMAC2 (10.8 mg·kg^{-1}; D) into 1 rat.
